# Tenfold Metalation of Ferrocene: Synthesis, Structures, and Metallophilic Interactions in FeC_10_(HgX)_10_

**DOI:** 10.1002/chem.202100261

**Published:** 2021-02-22

**Authors:** Susanne Margot Rupf, Gabriel Schröder, Robin Sievers, Moritz Malischewski

**Affiliations:** ^1^ Institute of Chemistry and Biochemistry Freie Universität Berlin Fabeckstr. 34–36 14195 Berlin Germany

**Keywords:** C−H activation, mercury, metalation, metallocenes, metal–metal interactions

## Abstract

The permercuration of ferrocene was achieved by reacting ferrocene with 10 equivalents of mercury(II) butyrate Hg(O_2_CC_3_H_7_)_2_ in a facile one‐pot reaction in multi‐gram scale and high yields. The butyrate groups in FeC_10_(HgX)_10_ (X=O_2_CC_3_H_7_) can be exchanged by treatment with trifluoro‐ or trichloroacetic acid (X=O_2_CCF_3_, O_2_CCCl_3_). Substitution of the trifluoroacetate groups by halides (X=Cl, F) proceeds easily in aqueous THF. The completeness of metalation was confirmed by NMR and vibrational spectroscopy, mass spectrometry, as well as elemental analysis. Additionally, the first crystal structures of permetallated metallocenes are presented: FeC_10_(HgX)_10_ (X=Cl, O_2_CCF_3_, O_2_CCCl_3_).

## Graphical abstract

**That's so metal**: All ten C−H bonds of ferrocene can be metalated in a simple one‐pot reaction by using mercury butyrate to give permercurated ferrocene, an air‐ and water stable compound. The completeness of metalation is confirmed by NMR and vibrational spectroscopy, mass spectrometry, as well as elemental analysis.

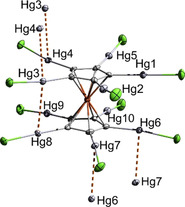

The functionalization of unreactive C−H bonds is a constant challenge in organometallic chemistry due to their high intrinsic stability.^[1]^ Typically, noble metal complexes are used for the activation of C−H bonds.^[2]^ Unfortunately, these metals are scarce and expensive,^[3]^ and commonly the reactivity of these metal complexes has to be tuned by sophisticated ligands. In the past years, efforts have intensified to use cheaper and more abundant 3d metals for C−H activation.^[4]^ However, in many cases C−H activation relies on the presence of directing groups.^[5]^ One of the most active metals in C−H activation, although nowadays widely ignored, is mercury. Mono‐ and polymercuration of aromatic compounds,[^6^, ^7^, ^8^, ^9^, ^10^, ^11^] olefins,^[12]^ as well as alkanes[^13^, ^14^] have been observed in reactions involving sources of Hg^2+^. Even unreactive C−H bonds such as methane can be brought to reaction using Hg(NTf_2_)_2_.^[14]^ However, interest in organomercury chemistry has declined during the past decades due to the high toxicity of organomercury compounds. Nevertheless, the utility of such mercurations can be demonstrated by the reaction of ferrocene with mercury(II) carboxylates.

Ferrocene derivatives have found numerous applications, for example, in material science, medicinal chemistry, and catalysis, which can be explained by its unusual stability towards moisture and oxygen as well as its unique redox properties.^[15]^ Consequently, functionalization of the C−H bonds in ferrocene is an active field of research.^[16]^ Although one‐pot reactions for the mono‐ and dilithiation of ferrocene are well established,[^17^, ^18^] higher degrees of metalation are difficult to achieve. For instance, refluxing a solution of ferrocene with eight equivalents of *n*BuLi for four days and subsequent quenching with D_2_O yields only to relatively low degrees of deuteration (FeC_10_D_*n*_H_10−*n*_: main products: *n*=2, 3, 4). The degree of lithiation can be slightly improved by addition of tetramethylethylenediamine (TMEDA).^[16]^ Mulvey and co‐workers have shown the use of strong, bimetallic bases for the tetrametalation of ferrocene.[^19^, ^20^, ^21^, ^22^] However, pioneering works of Winter and co‐workers demonstrated that much higher degrees of metalation could be accessed by mercuration of ferrocene, ruthenocene, and osmocene using mercury(II) carboxylates.[^23^, ^24^, ^25^, ^26^] Mercuration of aromatic systems by Hg^2+^ proceeds mechanistically via an electrophilic substitution pathway.^[27]^ Surprisingly, the reaction seems to proceed even faster with increasing degree of mercuration.^[28]^ Unfortunately, the insolubility of Winter's highly mercurated metallocenes prevented their full spectroscopic characterization. Moreover, the permercuration of ferrocene seemed also to be incomplete.^[29]^ In a review article, it was mentioned that the solubility and the degree of mercuration could be improved by replacing mercury(II) acetate by mercury(II) butyrate, but details were never published.^[7]^ Although organomercury compounds are highly toxic, they are valuable starting materials for functionalization or transmetalation reactions.[^30^, ^31^, ^32^] Therefore, we reinvestigated the permercuration of ferrocene with modern spectroscopic techniques.

FeC_10_(HgO_2_CC_3_H_7_)_10_ (**2**) was synthesized by reaction of ferrocene with mercury(II) butyrate (**1**) in 1,2‐dichloroethane (DCE) under reflux (Scheme 1). The substitution of the butyrate groups was performed by reaction of **2** with trifluoro‐ or trichloroacetic acid in THF yielding compounds FeC_10_(HgO_2_CCF_3_)_10_ (**3 a**) and FeC_10_(HgO_2_CCCl_3_)_10_ (**3 b**), respectively. The trifluoroacetate derivative **3 a** could be converted to the insoluble halide derivatives FeC_10_(HgX)_10_ (**4**) (X=F, Cl) by reaction of NaF/NaCl in aqueous THF mixtures. In contrast to the literature‐known permercurated metallocenes, compounds **2** and **3** are soluble in DMSO and tetrahydrothiophene (THT). THF adducts of **3** are even soluble in dichloromethane and methanol. This makes these compounds attractive starting materials for further reactions.

**Scheme 1:**
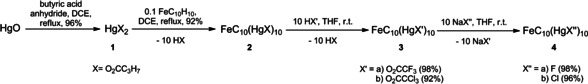
Synthesis of permetalated ferrocene derivatives FeC_10_(HgX)_10_ starting from mercury(II) oxide and substitution of the butyrate groups (X) by trifluoro‐ and trichloroacetate (X’) as well as halides (X’’).

Compounds **1**, **3 a**, **3 b**, and **4 b** were characterized via single crystal XRD. A detailed description of the crystal structure of mercury(II) butyrate Hg(O_2_CC_3_H_7_)_2_ **1** can be found in the Supporting Information. Single crystals of compounds **3 a**⋅4 THF⋅2 Et_2_O and **3 b**⋅10 THF⋅Et_2_O were obtained by diffusion of pentane into solutions of **3 a** and **3 b** in THF/Et_2_O mixtures, respectively. The permercurated metallocene with trifluoroacetate groups **3 a** (X=O_2_CCF_3_) crystallizes in monoclinic space group *P*2_1_/*n* and **3 b** (X=O_2_CCl_3_) in orthorhombic space group *Pbca*. The structures are shown in Figure 1 A, B as well as in the Supporting Information. The asymmetric units of both compounds contain a [FeC_5_(HgO_2_CCX_3_)_5_] unit. Due to a center of inversion located at the iron atom the overall formula in both cases is FeC_10_(HgO_2_CCX_3_)_10_. A staggered conformation of the two permercurated cyclopentadienyl rings is observed. The cyclopentadienyl‐iron distances are similar to ferrocene (Table 1).^[33]^ In both cases, the Cp rings are parallel (tilt angle 0°). In both structures all mercury atoms are coordinated by one Cp carbon atom and one carboxylate ligand. The coordination sphere is almost linear along the C−Hg−O axis. The distortion from linearity is caused by additional Hg−O contacts with carboxylate groups [2.857(8)–3.121(6) Å] and solvent molecules [2.539(6)–2.826(5) Å], all shorter than the sum of the van der Waals radii of oxygen (*r*_vdW_=1.54 Å^[34]^) and mercury (*r*_vdW_=1.75 Å^[35]^). Similar Hg−O(solvent) interactions have been reported previously.[^36^, ^37^, ^38^] Hg−Hg contacts shorter than twice the van‐der‐Waals radius of mercury are not observed. Taking all interactions into account the overall coordination number of the mercury atoms is four or five.

**Figure 1:**
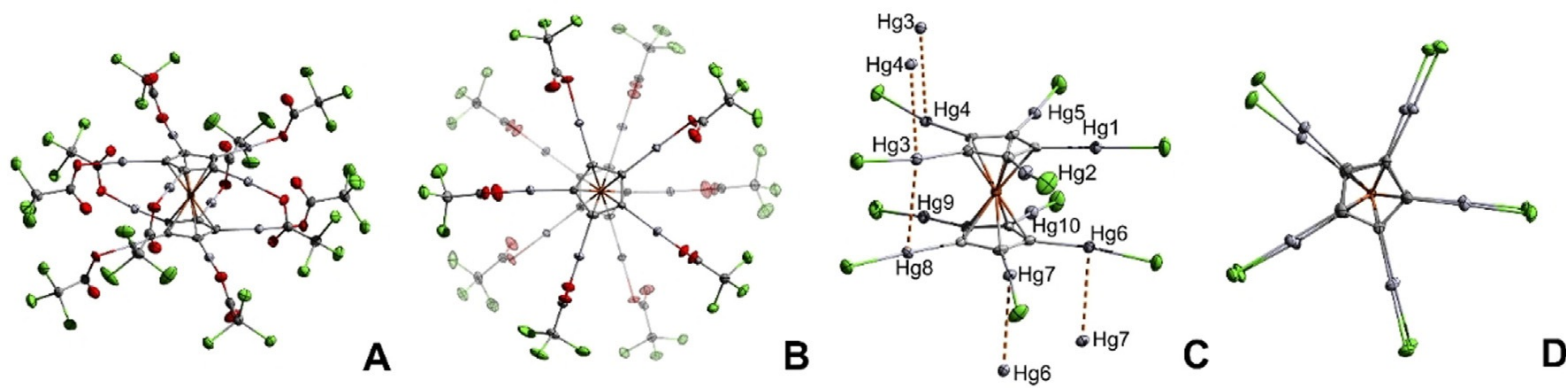
Molecular structure in solid state of permercurated ferrocene derivatives with trichloroacetate substituents (A, B) and chloride ligands (C, D) in different perspectives. Solvent molecules are omitted for clarity. Hg−Hg contacts are assigned as dashed lines. Ellipsoids are depicted with a 50 % probability level. Color code: light grey—mercury, orange—iron, green—chlorine, red—oxygen, grey—carbon.

**Table 1:** Selected bond lengths [Å] and angles [°].

Compound	**3 a**	**3 b**	**4 b**
Fe−Cp^center^	1.660(1)	1.657(1)	1.651(1), 1.658(1)
Hg−C	2.034(9)–2.040(8)	2.014(6)–2.037(6)	2.022(10)–2.046(9)
Hg−X^[a]^	2.082(7)–2.115(6)	2.082(4)–2.105(5)	2.326(2)–2.342(3)
Hg−Hg^intramol.^	3.543(1)–3.748(1)	3.668(1)–3.878(1)	3.447(1), 3.517(1)–3.637(1)
Hg−Hg^intermol.^	–	–	3.353(1), 3.369(1)
C−Hg−X^[a]^	168.78(31)–176.47(27)	174.15(21)–178.93(22)	170.78(21)–176.72(22)
Cp−Cp^tilt angle^	0	0	1.53(30)

[a] In case of **3 a** and **3 b**: X=O; **4 b**: X=Cl.

By serendipity, crystals of insoluble FeC_10_(HgCl)_10_ (**4 b**) were found in a decomposed sample of the trichloroacetate (**3 b**). **4 b**⋅9 DMSO crystallizes in the triclinic space group *P* $\bar{1}$ . In contrast to **3 a** and **3 b** the center of inversion is located outside of the metallocene moiety. Therefore, the asymmetric unit contains the whole molecule. All mercury atoms exhibit a distorted linear symmetry along the C−Hg−Cl axis, which is again a result of Hg−O(solvent) contacts of 2.708(6)–3.119(6) Å. A remarkable feature of the crystal structure is the presence of significant intra‐ and intermolecular mercurophilic Hg^+II^−Hg^+II^ interactions (Table 1). In contrast to the crystal structures of **3 a** and **3 b** an eclipsed conformation is observed for the two permercurated cyclopentadienyl rings (Figure 1 D). This different conformation could be a result of an intramolecular Hg^+II^−Hg^+II^ interaction between the atoms Hg3 and Hg8 of 3.447(1) Å (Figure 1 C), which is in accordance with other examples in the literature for Hg^+II^−Hg^+II^ interactions. Interestingly, the Cp rings are not completely parallel (tilt angle 1.6°) which might be related to this d^10^–d^10^ interaction. Additionally, intermolecular interactions between Hg3 and Hg4 as well as Hg6 and Hg7 of 3.353(1) and 3.369(1) Å are observed, which are even shorter than the intramolecular interaction. As a consequence, no isolated ferrocene moieties are observed in the solid‐state structure, but a polymeric chain of ferrocene units connected by Hg−Hg contacts (Figures S7 and S8), which could explain the insolubility of the compound. In contrast to this, the well soluble derivatives **3** (trihaloacetate) exhibit no significant Hg^+II^−Hg^+II^ contacts. Taking all interactions into account the coordination number of Hg4, Hg6, and Hg7 is five and of Hg3 and Hg8 six for FeC_10_(HgCl)_10_ (**4 b**). At this point it should be noted that short Hg^+II^−Hg^+II^ interactions are usually found when the coordination number of the metal center is small.^[39]^ Therefore, the finding of rather short crystallographically independent Hg^+II^−Hg^+II^ contacts is surprising, since the coordination number of the mercury atoms is relatively high. So far, examples of strong Hg^+II^−Hg^+II^ interactions are relatively rare.[^40^, ^41^, ^42^] Only a small number of crystal structures contain shorter contacts than the here reported 3.353(1) Å.[^36^, ^43^, ^44^, ^45^, ^46^, ^47^] Furthermore, only a few examples of crystal structures of permercurated compounds are known so far.[^48^, ^49^, ^50^]

The soluble compounds **2** (butyrate) and **3** (trihaloacetate) were characterized via NMR spectroscopy. The ^13^C NMR spectra of **3 a** and **3 b** display three signals, respectively. Compound **2** shows five signals. (Figure 2). The most downfield shifted signals chemical shifts (**2**: 177.5, **3 a**: 164.2, **3 b**: 167.8 ppm) can be assigned to the carbonyl groups. The signals for the cyclopentadienyl rings can be found at (**2**: 97.8, **3 a**: 97.4, **3 b**: 97.1 ppm), which is similar to those signals of other polymercurated ferrocene derivatives.[^38^, ^51^] All remaining signals correspond to the alkyl groups of the carboxylates. The fact that only one Cp−C signal is visible for all three compounds confirms the highly symmetric metallocene structure. Furthermore, no Cp−H signals can be found in the ^1^H NMR spectra (see the Supporting Information). Unfortunately, neither a signal in the ^199^Hg NMR spectrum nor ^199^Hg satellites for the Cp−C signals in the ^13^C NMR spectrum could be observed, which is probably a consequence of ^199^Hg line broadening due to chemical shift anisotropy.[^52^, ^53^, ^54^, ^55^] Elemental analysis of the permercurated ferrocenes matches the expected compositions. Electron spray ionization (ESI)‐MS shows a peak at *m*/*z=*3053.05, which can be unambiguously assigned to [FeC_10_(HgO_2_CC_3_H_7_)_10_]^+^ (calculated: *m*/*z=*3053.08). Hence, we conclude that the ten‐fold electrophilic substitution of ferrocene by mercury(II) butyrate was successful.

**Figure 2:**
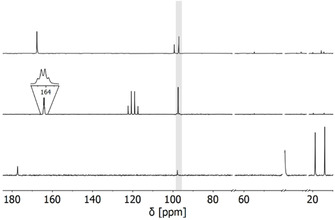
^13^C NMR spectra of FeC_10_(HgO_2_CC_3_H_7_)_10_ (**2**) (bottom, 176 MHz, [D_6_]DMSO, r.t.), FeC_10_(HgO_2_CCF_3_)_10_ (**3a**) (middle, 176 MHz, [D_8_]THF, r.t.), and FeC_10_(HgO_2_CCCl_3_)_10_ (**3b**) (top, 176 MHz, [D_8_]THF, r.t.). Signals of the deuterated solvents are omitted for clarity. Cp−C signals are highlighted in grey.

While the IR spectra of all permercurated metallocenes show only bands which correspond to the mercury‐bound carboxylates (see the Supporting Information), Raman spectra contain more information especially with respect to Hg−Cp bonds (Figure 3). The bands between 930 and 960 cm^−1^ are present in all spectra which is characteristic for the metallocene backbone and correspond to the vibration of the cyclopentadienyl rings. The bands between 100 and 120 cm^−1^ correspond to Hg−C stretch vibrations. Other bands are associated to vibrations of the Hg‐bonded substituents (X). The frequencies of the Hg−X bonds between 300 and 500 cm^−1^ are similar to the analogous vibrations in HgX_2_.[^56^, ^57^]

**Figure 3:**
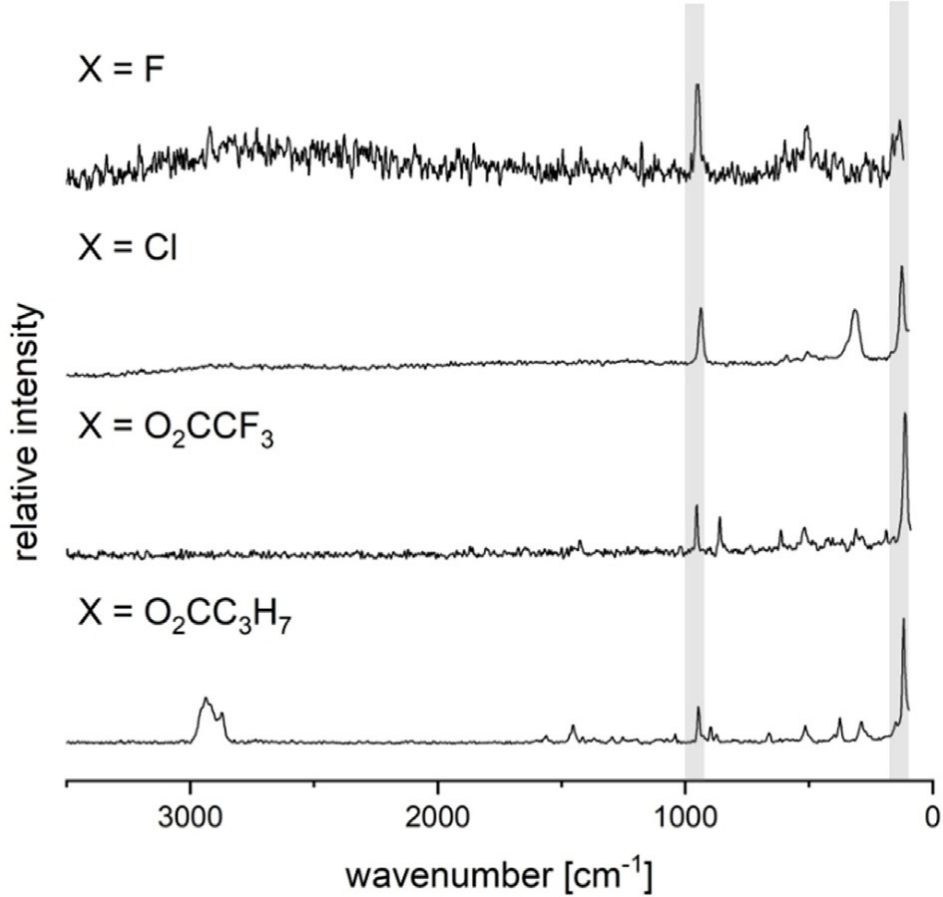
Raman spectra (1064 nm) of FeC_10_(HgX)_10_ derivatives. Characteristic bands for the permercurated metallocene moiety are highlighted in grey.

Cyclovoltammetric measurements of the permercurated ferrocene derivatives only revealed one irreversible oxidation process for the trifluoroacetate **3 a** at *E*_p_=+0.87 V in THF (Figure S38) while the less stable trichloroacetate **3 b** visibly decomposed during the measurements under formation of insoluble material. The butyrate derivative **2** did not have enough solubility in THF. When tetrahydrothiophene was used instead to increase the solubility, no oxidation process was observed in the corresponding electrochemical window.

In summary, we demonstrated the synthesis of ten‐fold metallated ferrocene derivatives FeC_10_(HgX)_10_ with mercury(II) carboxylate (X=O_2_CC_3_H_7_, O_2_CCF_3_, O_2_CCCl_3_) and halide substituents (X=F, Cl). These compounds can be prepared in facile one‐pot reactions in multi‐gram scale and high yields without the need for inert conditions. The complete metalation as well as the purity of the samples was demonstrated by a variety of spectroscopic methods. Additionally, we present the first crystal structures of permercurated metallocenes. FeC_10_(HgO_2_CCF_3_)_10_, FeC_10_(HgO_2_CCCl_3_)_10_, and FeC_10_(HgCl)_10_. Depending on the substituent, different conformers are observed in the solid‐state structures. The permercurated ferrocenes with carboxylate groups show good solubility in organic solvents but no significant Hg−Hg interactions. In contrast, FeC_10_(HgCl)_10_ is completely insoluble. Its crystal structure displays relatively short intra‐ and intermolecular Hg^+II^−Hg^+II^ contacts.

Beside their aesthetic appearance, the permercurated ferrocenes might be very useful starting materials for further functionalization or transmetalation reactions. From a more general point of view, one can only be amazed about the extreme reactivity of Hg^2+^ in C−H functionalization reactions. However, it might be worthwhile to further investigate this unique behavior with the hope of finding ways to mimic this reactivity with other, less toxic elements.

## Conflict of interest

The authors declare no conflict of interest.

## Supporting information

As a service to our authors and readers, this journal provides supporting information supplied by the authors. Such materials are peer reviewed and may be re‐organized for online delivery, but are not copy‐edited or typeset. Technical support issues arising from supporting information (other than missing files) should be addressed to the authors.

Supplementary

## References

[1] R. G. Bergman , Nature 2007, 446, 391–393. 17377575 10.1038/446391a

[2] N. Kuhl , M. N. Hopkinson , J. Wencel-Delord , F. Glorius , Angew. Chem. Int. Ed. 2012, 51, 10236–10254; 10.1002/anie.201203269 22996679

[3] E. Nakamura , K. Sato , Nat. Mater. 2011, 10, 158–161. 21336288 10.1038/nmat2969

[4] P. Gandeepan , T. Müller , D. Zell , G. Cera , S. Warratz , L. Ackermann , Chem. Rev. 2019, 119, 2192–2452. 30480438 10.1021/acs.chemrev.8b00507

[5] C. Sambiagio , D. Schönbauer , R. Blieck , T. Dao-Huy , G. Pototschnig , P. Schaaf , T. Wiesinger , M. F. Zia , J. Wencel-Delord , T. Besset , B. U. W. Maes , M. Schnürch , Chem. Soc. Rev. 2018, 47, 6603–6743. 30033454 10.1039/c8cs00201k PMC6113863

[6] M. R. Haneline , R. E. Taylor , F. P. Gabbaï , Chem. Eur. J. 2003, 9, 5188–5193.

[7] C. H. Winter , K. N. Seneviratne , A. Bretschneider-Hurley , Comm. Inorg. Chem. 1996, 19, 1–23.

[8] A. Grirrane , I. Resa , D. del Río , A. Rodríguez , E. Álvarez , K. Mereiter , E. Carmona , Inorg. Chem. 2007, 46, 4667–4676. 17461576 10.1021/ic0624672

[9] G. B. Deacon , G. J. Farquharson , J. Organomet. Chem. 1974, 67, C1–C3.

[10] R. M. Harrison , T. Brotin , B. C. Noll , J. Michl , Organometallics 1997, 16, 3401–3412.

[11] M. Bausch , M. Vogel , H. Rosenberg , J. Org. Chem. 1957, 22, 900–903.

[12] A. K. Brisdon , I. R. Crossley , R. G. Pritchard , Organometallics 2005, 24, 5487–5490.

[13] B. Korpar-Čolig , Z. Popović , D. Matković-Čalogović , Organometallics 1993, 12, 4708–4713.

[14] N. J. Gunsalus , S. H. Park , B. G. Hashiguchi , A. Koppaka , S. J. Smith , D. H. Ess , R. A. Periana , Organometallics 2019, 38, 2319–2322.

[15] D. Astruc , Eur. J. Inorg. Chem. 2017, 6–29.

[16] L. A. López , E. López , Dalton Trans. 2015, 44, 10128–10135. 25973598 10.1039/c5dt01373a

[17] F. Rebiere , O. Samuel , H. B. Kagan , Tetrahedron Lett. 1990, 31, 3121–3124.

[18] J. J. Bishop , A. Davison , M. L. Katcher , D. W. Lichtenberg , R. E. Merrill , J. C. Smart , J. Organomet. Chem. 1971, 27, 241–249.

[19] W. Clegg , K. W. Henderson , A. R. Kennedy , R. E. Mulvey , C. T. O'Hara , R. B. Rowlings , D. M. Tooke , Angew. Chem. Int. Ed. 2001, 40, 3902–3905; 11668570

[20] P. C. Andrikopoulos , D. R. Armstrong , W. Clegg , C. J. Gilfillan , E. Hevia , A. R. Kennedy , R. E. Mulvey , C. T. O'Hara , J. A. Parkinson , D. M. Tooke , J. Am. Chem. Soc. 2004, 126, 11612–11620. 15366908 10.1021/ja0472230

[21] W. Clegg , E. Crosbie , S. H. Dale-Black , E. Hevia , G. W. Honeyman , A. R. Kennedy , R. E. Mulvey , D. L. Ramsay , S. D. Robertson , Organometallics 2015, 34, 2580–2589.

[22] G. W. Honeyman , D. R. Aarmstrong , W. Clegg , E. Hevia , A. R. Kennedy , R. McLellan , S. A. Orr , J. A. Parkinson , D. L. Ramsay , S. D. Robertson , S. Towie , R. E. Mulvey , Chem. Sci. 2020, 11, 6510–6520. 10.1039/d0sc01612h PMC8152701 34094116

[23] C. H. Winter , Y.-H. Han , R. L. Ostrander , A. L. Rheingold , Angew. Chem. Int. Ed. Engl. 1993, 32, 1161–1163;

[24] Y.-H. Han , M. J. Heeg , C. H. Winter , Organometallics 1994, 13, 3009–3019.

[25] S. A. Kur , C. H. Winter , J. Organomet. Chem. 1996, 512, 39–44.

[26] A. F. Neto , A. D. L. Borges , I. P. de Arruda Campos , J. Miller , Synth. React. Inorg. Met.-Org. Chem. 1997, 27, 1543–1551.

[27] J. A. F. Cunningham , Organometallics 1997, 16, 1114–1122.

[28] C. H. Winter , Y.-H. Han , M. J. Heeg , Organometallics 1992, 11, 3169–3171.

[29] S. A. Kur , A. L. Rheingold , C. H. Winter , Inorg. Chem. 1995, 34, 414–416.

[30] M. Olaru , R. Kather , E. Hupf , E. Lork , S. Mebs , J. Beckmann , Angew. Chem. Int. Ed. 2018, 57, 5917–5920; 10.1002/anie.201712944 29527798

[31] M. Olaru , S. Krupke , E. Lork , S. Mebs , J. Beckmann , Dalton Trans. 2019, 48, 5585–5594. 30942221 10.1039/c9dt00827f

[32] S. Furan , E. Lork , S. Mebs , E. Hupf , J. Beckmann , Z. Anorg. Allg. Chem. 2020, 646, 856–865.

[33] P. Seiler , J. D. Dunitz , Acta Crystallogr. 1979, 35, 1068–1074.

[34] A. Bondi , J. Phys. Chem. A 1964, 68, 441–451.

[35] P. Pyykkö , M. Straka , Phys. Chem. Chem. Phys. 2000, 2, 2489–2493.

[36] H. Schmidbaur , H.-J. Öller , D. L. Wilkinson , B. Huber , G. Müller , Chem. Ber. 1989, 122, 31–36.

[37] M. Tschinkl , A. Schier , J. Riede , F. P. Gabbaï , Angew. Chem. Int. Ed. 1999, 38, 3547–3549; 10.1002/(sici)1521-3773(19991203)38:23<3547::aid-anie3547>3.0.co;2-p 10602238

[38] K. Venkatasubbaiah , J. W. Bats , A. L. Rheingold , F. Jäkle , Organometallics 2005, 24, 6043–6050.

[39] J. Echeverría , J. Cirera , S. Alvarez , Phys. Chem. Chem. Phys. 2017, 19, 11645–11654. 28435953 10.1039/c7cp00542c

[40] J. B. King , M. R. Haneline , M. Tsunoda , F. P. Gabbaï , J. Am. Chem. Soc. 2002, 124, 9350–9351. 12167011 10.1021/ja0268534

[41] M. R. Haneline , F. P. Gabbaï , Angew. Chem. Int. Ed. 2004, 43, 5471–5474; 10.1002/anie.200461152 15484255

[42] M. A. Omary , R. M. Kassab , M. R. Haneline , O. Elbjeirami , F. P. Gabbaï , Inorg. Chem. 2003, 42, 2176–2178. 12665343 10.1021/ic034066h

[43] U. Patel , H. B. Singh , G. Wolmershäuser , Angew. Chem. Int. Ed. 2005, 44, 1715–1717; 10.1002/anie.200462487 15693040

[44] E. Hupf , E. Lork , S. Mebs , J. Beckmann , Inorg. Chem. 2015, 54, 1847–1859. 25612107 10.1021/ic502728v

[45] R. Galassi , F. Bachechi , A. Burini , J. Mol. Struct. 2006, 791, 82–88.

[46] P. D. Harvey , K. T. Aye , K. Hierso , E. Isabel , I. Lognot , Y. Mugnier , F. D. Rochon , Inorg. Chem. 1994, 33, 5981–5982.

[47] N. L. Pickett , O. Just , D. G. VanDerveer , W. S. Rees Jr , Acta Crystallogr. 2000, C56, 412–413. 10.1107/S0108270199016339 10815189

[48] D. Grdenić , M. Sikirica , B. Korpar-Čolig , J. Organomet. Chem. 1978, 153, 1–7.

[49] D. Grdenić , B. Kamenar , B. Korpar-Čolig , M. Sikirca , G. Jovanovski , J. Chem. Soc. Chem. Commun. 1974, 646–647.

[50] D. Grdenić , M. Sikirica , D. Matković-Čalogović , J. Organomet. Chem. 1986, 306, 1–7.

[51] V. Sathesh , R. V. G. N. Chinta , R. Mamidala , V. Mukundam , K. Dhanunjayarao , K. Venkatasubbaiah , J. Organomet. Chem. 2017, 853, 74–80.

[52] R. E. Wasylishen , R. E. Lenkinski , C. Rodger , Can. J. Chem. 1982, 60, 2113–2117.

[53] J. G. Melnick , K. Yurkerwich , D. Buccella , W. Sattler , G. Parkin , Inorg. Chem. 2008, 47, 6421–6426. 18533648 10.1021/ic8005426

[54] M. Maliarik , I. Persson , Magn. Reson. Chem. 2005, 43, 835–842. 16025553 10.1002/mrc.1625

[55] R. Benn , H. Günther , A. Maercker , V. Menger , P. Schmitt , Angew. Chem. Int. Ed. Engl. 1982, 21, 295–296;

[56] R. P. J. Cooney , J. R. Hall , J. Inorg. Nucl. Chem. 1972, 34, 1519–1527.

[57] A. J. Downs , E. A. V. Ebsworth , H. J. Emeléus , J. Chem. Soc. 1962, 1254–1260.

[58] Deposition numbers 2047737 (for FeC_10_(HgCl)_10_), 2047740 (for FeC_10_(HgO_2_CCCl_3_)_10_), and 2047742 (for FeC_10_(HgO_2_CCF_3_)_10_) contain the supplementary crystallographic data for this paper. These data are provided free of charge by the joint Cambridge Crystallographic Data Centre and Fachinformationszentrum Karlsruhe Access Structures service.

