# Supplementary material for: Tenfold Metalation of Ferrocene: Synthesis, Structures, and Metallophilic Interactions in FeC10(HgX)10
Source: Chemistry. 2021 Feb 22;27(16):5125–9. doi: 10.1002/chem.202100261 (PMC8048484; doi:10.1002/chem.202100261)
Supplement: Supplementary file 1 — Supplementary [file CHEM-27-5125-s001.pdf]

Chemistry–A European Journal

Supporting Information

Tenfold Metalation of Ferrocene: Synthesis, Structures, and Metallophilic Interactions in $FeC_{10}(HgX)_{10}$

Susanne Margot Rupf, Gabriel Schröder, Robin Sievers, and Moritz Malischewski*[a]

SUPPORTING INFORMATION

Table of Contents

Experimental Procedures S1
General Procedures S1
Synthesis S1
Crystallographic Data S5
References S10
Appendix S11

Experimental Procedures

General Procedures

CAUTION: Mercury compounds are highly toxic. Therefore, the chemicals should be handled under a well-ventilated fume hood. Great care must be taken that organomercury compounds are not spilled on the skin. It is advisable to use multiple layers of impenetrable gloves and to remove the outer layer immediately if material is spilled on it. The face might be protected against splashes by using a face shield. Due to the hazardous effects of mercury on the environment, great care must be taken to prevent contamination of the wastewater with mercury.

Commercially available chemicals were used as received, unless otherwise noted. All manipulations were performed in air. Yellow and red mercury oxide as well as 1,2-dichloroethane (carcinogenic) was purchased from abcr GmbH. Sodium chloride was purchased from Carl Roth GmbH + Co. KG, sodium fluoride from Riedel de Haen AG, butyric acid anhydride from Sigma-Aldrich, 1,1,1-trifluoroacetic acid from Fluorochem, 1,1,1-trichloroacetic acid from Merck KGaA, and ferrocene from Alfa Aesar.

^{1}H, ^{13}C and ^{19}F NMR spectra were recorded on a Bruker AVANCE III 700 spectrometer by using 6mm NMR glass tubes. All reported chemical shifts (δ) are referenced to the Ξ values given in IUPAC recommendations of 2008 using the ^{2}H signal of the deuterated solvent as internal reference.[1] All chemical shifts (δ) are given in parts per million (ppm) and the signals are specified according to the multiplicity (s = singlet, d = doublet, t = triplet, q = quartet, m = multiplet, br = broad) and the coupling constants *J* in Hz. Mass spectrometry was performed on a Agilent 6210 ESI TOF (electrospray ionization time-of-flight spectrometer) of Aglient Technologies, Santa Clara, CA in negative mode. Flow rates were set to 10 $\mu L \cdot min^{-1}$. The evaluation of the resulting data occurred by using Mmass 5.5.0.[2] The program MestRe Nova Version 14.0.1 was used to evaluate and plot the data.[3]

Infrared spectra were measured using a Thermo-Scientific Nicolet iS10 FTIR spectrometer with DuraSamplIR accessory in attenuated total reflection at room temperature. Raman spectra were recorded on a Bruker MultiRAM II equipped with a low-temperature Ge detector (1064 nm, 30-80 mW, resolution 2 cm^{-1}). The software OriginPro 2017G was used to plot the data.[4]

Cyclic voltammetry was performed on a Interface 1010 B Potentiostat/Galvanostat/ZRA from Gamry Instruments. The investigations were carried out starting from 0 V going to the oxidation first and then to the reduction. The measurements were performed at a scan rate of 100 mV/s in anhydrous solvents under argon atmosphere using tetrabutylammonium hexafluorophosphate as the supporting electrolyte and platinum wires as working-, counter-, and quasi-reference electrodes. The voltammograms were internally referenced against $Fc^{0/+}$. The software OriginPro 2017G was used to plot the data.[4] Anhydrous THF was stored in Young flasks under argon atmosphere over molar sieve (3 Å) which was dried beforehand at 250 °C under high vacuum. The conducting salt was dried at 250 °C under high vacuum. The solvents were condensed on the conducting salt in the cyclic voltammetry cells via a vacuum line.

X-Ray data were collected on a BRUKER D8 Venture system. Data were collected at 100(2) K using graphite-monochromated Mo K_{α} radiation (λ_{α} = 0.71073 Å). The strategy for the data collection was evaluated by using the Smart software. The data were collected by the standard "*ψ-ω* scan techniques" and were scaled and reduced using Saint+software. The structures were solved by using Olex2,[5] the structure was solved with the XT[6] structure solution program using Intrinsic Phasing and refined with the XL refinement package[7,8] using Least Squares minimization. If it is noted, bond length and angles were measured with Diamond Crystal and Molecular Structure Visualization Version 4.6.2.[9] Drawings were generated with POV-Ray.[10] Deposition numbers CCDC 2047737 ($FeC_{10}(HgCl)_{10}$), 2047740 ($FeC_{10}(HgO_2CCCl_3)_{10}$) and 2047742 ($FeC_{10}(HgO_2CCF_3)_{10}$) contains the supplementary crystallographic data for this paper. These data are provided free of charge by the joint Cambridge Crystallographic Data Centre and Fachinformationszentrum Karlsruhe Access Structures service www.ccdc.cam.ac.uk/structures.

Synthesis

Bis(butyryloxy)mercury(II) – "Mercury(II) butyrate" ***1***

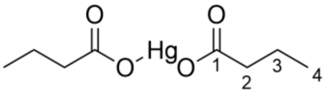

Butyric acid anhydride (18.5 mL, 113 mmol, 1.1 eq.) and 1,2-dichloroethane (600 mL) were placed in a 1000 mL round bottom flask. Mercury(II)oxide (22.2 g, 103 mmol, 1.0 Eq.) was added and the suspension was stirred under reflux for 12 h (Note: yellow HgO dissolves faster than red HgO). After cooling to room temperature the product was obtained as a colourless, crystalline solid. The

precipitate was filtered off and washed with 1,2-dichloroethane (400 mL) and *n*-pentane (600 mL). The residue was dried in high vacuum to afford compound **1** (37.2 g, 96%) as a colorless, crystalline solid.

^{1}H NMR (400 MHz, dmso-d$_6$, r.t.): *δ* [ppm] = 3.04 (t, *J* = 7.4 Hz, 2H), 2.39 (qt, *J* = 7.4, 7,4 Hz, 2H), 1.71 (t, *J* = 7.4 Hz, 3H).

^{13}C{^{1}H} NMR (101 MHz, dmso-d$_6$, r.t.): *δ* [ppm] = 191.2 (s, C1), 50.5 (s, C2), 34.4 (s, C3), 28.3 (s, C4).

FT-IR (ATR): $\tilde{\nu}$ [cm^{-1}] = 2964 (ν CH$_3$), 2932 (ν CH$_2$), 2871 (ν CH$_2$), 1600 (ν_s COO), 1572 (ν_{as} COO), 1465 (δ CH$_2$), 1373 (δ CH$_3$), 1327, 1290, 1240, 1121, 1100, 896, 756, 727 (δ CH$_2$).

Raman (1064 nm, r.t.): $\tilde{\nu}$ [cm^{-1}] = 2971 (ν CH$_3$), 2938 (ν CH$_2$), 2880 (ν CH$_2$), 1606 (ν_{sym} COO), 1449 (ν_{asym} COO)

MS (ESI+, r.t.): [Hg(O$_2$C$_4$H$_7$)$_2$ + Na]$^+$ calculated: 399.05, found: 399.05.

Elemental analysis: m [%] = calculated: N: 0.00, C: 25.64, H: 3.765, found: N: 0.01, C: 25.89, H: 3.817.

*Decakis(butyryloxymercury(II))ferrocene **2***

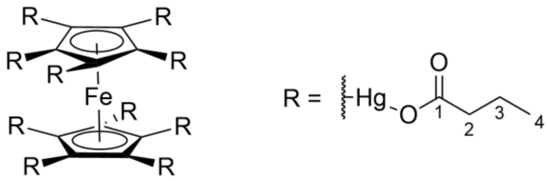

Mercury(II) butyrate (32.5 g, 86.7 mmol, 11 eq.) and 1,2-dichloroethane (600 mL) were placed in a 1000 mL round bottom flask. Ferrocene (1.47 g, 7.88 mmol, 1.0 Eq.) was added and the suspension was stirred under reflux for 18 h. The suspension was filtered while still hot and the precipitate was washed with 1,2-dichloroethane (300 mL) and *n*-pentane (400 mL). The solid was dried in high vacuum to afford compound **2** (22.1 g, 92%) as an orange, amorphous solid.

^{1}H NMR (700 MHz, dmso-d$_6$, r.t.) δ [ppm] = 2.11 (t, J = 7.4 Hz, 2H), 1.53 (qt, J = 7.4 Hz, 2H), 0.91 (t, J = 7.4 Hz, 3H).

^{13}C{^{1}H} NMR (176 MHz, dmso-d$_6$, r.t.) δ [ppm] = 177.5 (s, C1), 97.6 (s, C$_{Cp}$), 38.7 (s, C2), 18.9 (s, C3), 13.9 (s, C4).

FT-IR (ATR): $\tilde{\nu}$ [cm^{-1}] = 2960 (ν CH$_3$), 2931 (ν CH$_2$), 2872 (ν CH$_2$), 1610 (ν_s C=O), 1561 (s, ν_{as} C=O), 1368 (δ CH$_3$), 1338, 1241, 1216, 661.

Raman (1064 nm, r.t.): $\tilde{\nu}$ [cm^{-1}] = 2935 (ν CH$_3$), 2870 (ν CH$_2$), 1559, 1459, 950 (δ Cp), 892 (δ C-C), 869 (δ C-C), 664, 501, 377 (ν Hg-X), 285, 123 (ν Hg-Cp).

MS (ESI+, r.t.): [FeC$_{10}$(HgO$_2$C$_4$H$_7$)$_{10}$]$^+$ calculated: 3053.08, found: 3053.05.

Elemental analysis: m [%] = calculated: N: 0.00, C: 19.67, H: 2.31, found: N: 0.01, C: 20.37, H: 2.34.

*Decakis(trifluoroacetoxymercury(II))ferrocene **3a***

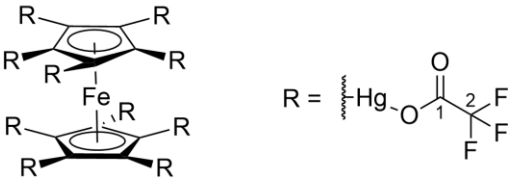

To a suspension of decakis(butyryloxymercury)ferrocene **2** (30 g, 9.83 mmol, 1 Eq.) in THF (50 mL) trifluoroacetic acid (ca. 10 mL) was added dropwise under vigorous stirring until the suspension became clear. The mixture was then concentrated under reduced pressure yielding a red solution. The product was precipitated by addition of *n*-pentane (500 mL) and filtered off. Afterwards the solid was washed with *n*-pentane and dichloromethane. This procedure was repeated three times. The product was obtained as an orange solid (31.98 g, 98%). Single crystals of FeC$_{10}$(HgO$_2$CCF$_3$)$_{10}$ · 4 THF · 2 OEt$_2$ were obtained by diffusion of *n*-pentane into a solution of compound **3a** in a mixture of THF and diethylether.

^{19}F NMR (564 MHz, THF-d_8, r.t.) δ [ppm] = −74.5 (s, CF_3).

$^{13}C\{^1H\}$ NMR (176 MHz, dmso-d_6, r.t.) δ [ppm] = 164.2 (q, *J* = 39.5 MHz, C1), 119.9 (q, *J* = 287.6 MHz, C2), 97.4 (s, C_{Cp}).

FT-IR (ATR): $\tilde{\nu}$ [cm^{-1}] = 1646 (ν_{asym} COO), 1429 (ν_{sym} COO), 1173 (ν C-C), 1144 (ν C-C), 862 (ν_{asym} CF_3), 794 (ν_{asym} CF_3), 733 (ν_{sym} CF_3).

Raman (1064 nm, r.t.): $\tilde{\nu}$ [cm^{-1}] = 1423 (ν_{asym} COO), 954 (δ Cp), 856 (δ C-C), 615 (δ C-F), 514 (δ C-F), 311 (ν Hg-X), 116 (ν Hg-Cp).

Elemental analysis: m [%] = calculated: N: 0.00, C: 10.88, H: 0.00, found: N: 0.01, C: 11.30, H: 0.01.

Decakis(trichloroacetoxymercury(II))ferrocene ***3b***

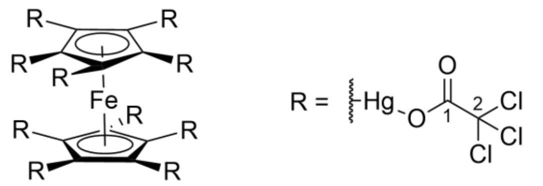

To a suspension of decakis(butyryloxymercury)ferrocene **2** (0.92 g, 0.31 mmol, 1 Eq.) in THF (10 mL) trichloroacetic acid (ca. 1 g) was added portion wise under vigorous stirring until the suspension became clear. THF was removed under reduced pressure yielding a red solid. The crude product was dissolved in 3 mL THF. Then, the solution was precipitated by addition of *n*-pentane (ca. 200 mL). Afterwards the precipitate was filtered off and washed with *n*-pentane and dichloromethane. This procedure was repeated three times. The product was obtained as an orange solid (1.09 g ,92%). Single crystals of $FeC_{10}(HgO_2CCCl_3)_{10}$ · 10 THF · OEt_2 were obtained by diffusion of *n*-pentane into a solution of compound **3b** in a THF/Et_2O mixture.

$^{13}C\{^1H\}$ NMR (176 MHz, dmso-d_6, r.t.) δ [ppm] = 167.8 (s, C1), 99.4 (s, C2), 97.2 (s, C_{Cp}).

FT-IR (ATR): $\tilde{\nu}$ [cm^{-1}] = 1640 (ν_{asym} COO), 1326 (ν_{sym} COO), 827 (ν_{asym} CCl_3), 751 (ν_{asym} CCl_3), 669 (ν_{sym} CCl_3).

Raman (1064 nm, r.t.): $\tilde{\nu}$ [cm^{-1}] = 1341 (ν_{asym} COO), 960 (δ Cp), 852 (δ C-C), 774 (ν_{asym} CCl_3), 441, 374 (ν Hg-O), 94 (ν Hg-Cp).

Elemental analysis: m [%] = calculated: N: 0.00, C: 9.47, H: 0.00, found: N: 0.01, C: 9.37, H: 0.01.

Decakis(fluoridomercury(II))ferrocene ***4a***

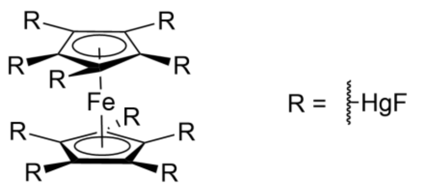

To solution of decakis(trifluoroacetoxymercury(II))ferrocene **3a** (1.40 g, 0.42 mmol) in THF (5 mL) sodium fluoride (200 mg, 4.76 mmol, 11 Eq.) was added. After addition of water (5 mL) a red precipitate formed. The precipitate was filtered off and washed with water, THF and dichloromethane. After drying in high vacuum, the product was obtained as a red solid (0.98 g, 98%).

Raman (1064 nm, r.t.): $\tilde{\nu}$ [cm-^{1}] = 947 (δ Cp), 507 (ν Hg-F) 122 (ν Hg-Cp).

Elemental analysis: m [%] = calculated: N: 0.00, C: 5.06, H: 0.00, found: N: 0.01, C: 5.16, H: 0.01.

Decakis(chloridomercury(II))ferrocene ***4b***

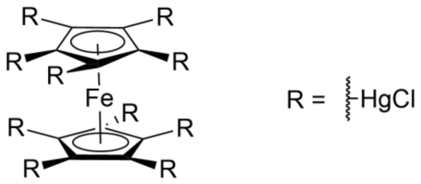

To solution of decakis(trifluoroacetoxymercury(II))ferrocene **3a** (1.40 g) in THF (5 mL) sodium chloride (300 mg, 5.17 mmol, 12 Eq.) was added. After addition of water (5 mL) a red precipitate formed. The precipitate was filtered off and washed with water, THF and dichloromethane. After drying in high vacuum, the product was obtained as a red solid (1.02 g, 96 %). Single crystals of $FeC_{10}(HgCl)_{10}$ · 9 DMSO formed upon decomposition of decakis(mercury(II) trichloroacetate)ferrocene in dimethylsulfoxide at room temperature over weeks.

Raman (1064 nm, r.t.): $\tilde{\nu}$ [cm^{-1}] = 937 (δ Cp), 318 (ν Hg-Cl), 119 (ν Hg-Cp).

Elemental analysis: m [%] = calculated: N: 0.00, C: 5.33, H: 0.00, found: N: 0.01, C: 5.53, H: 0.01.

Crystallographic Data

Table S1. Crystallographic data.

Compound	$FeC_{10}(HgO_2CCF_3)_{10}$ · 4 THF · 2 OEt_2	$FeC_{10}(HgO_2CCCl_3)_{10}$ · 10 THF · OEt_2	$FeC_{10}(HgCl)_{10}$ · 9 DMSO
**Empirical formula**	$C_{54}H_{52}F_{30}FeHg_{10}O_{26}$	$C_{74}H_{90}Cl_{30}FeHg_{10}O_{31}$	$C_{28}H_{54}Cl_{10}FeHg_{10}O_9S_9$
**Formula weight**	2748.70	4600.70	3239.50
**Temperature/K**	100.0	100.0	100.0
**Crystal system**	monoclinic	orthorhombic	triclinic
**Space group**	$P2_1/n$	Pbca	$P\bar{1}$
**a/Å**	10.2188(5)	22.3645(8)	15.6348(8)
**b/Å**	26.8902(12)	19.9277(6)	16.8576(9)
**c/Å**	14.8988(6)	26.6101(9)	17.3256(9)
**α/°**	90	90	105.992(2)
**β/°**	98.1234(15)	90	114.194(2)
**γ/°**	90	90	102.216(2)
**Volume/Å³**	4052.9(3)	11859.4(7)	3719.8(3)
**Z**	2	4	2
**ρ_{calc}/g·cm³**	3.072	2.577	2.892
**μ/mm^{-1}**	19.173	13.753	21.383
**F(000)**	3360.0	8472.0	2868.0
**Crystal size/mm³**	0.331 x 0.254 x 0.194	0.27 × 0.26 × 0.25	0.65 × 0.159 × 0.098
**Crystal shape**	block	cube	Needle
**Radiation**	MoKα (λ = 0.71073)	MoKα (λ = 0.71073)	MoKα (λ = 0.71073)
**2Θ range for data collection/°**	4.1 to 51.516	4.106 to 56.598	4.016 to 50.832
**Index ranges**	-12 ≤ h ≤ 12, -32 ≤ k ≤ 32, -17 ≤ l ≤ 18	- 29 ≤ h ≤ 29, -26 ≤ k ≤ 26, -35 ≤ l ≤ 35	-18 ≤ h ≤ 17, -20 ≤ k ≤ 20, -20 ≤ l ≤ 20
**Reflections collected**	116438	189726	180244
**Independent reflections**	7763 [R_{int} = 0.0623, R_{sigma} = 0.0257]	14717 [R_{int} = 0.0615, R_{sigma} = 0.0243]	13670 [R_{int} = 0.0511, R_{sigma} = 0.0221]
**Data/restrains/parameters**	7763/0/532	14717/0/700	13670/1/663
**Goodness-of-fit on F²**	1.119	1.127	1.033
**Final R indexes [I>=2σ(I)]**	R_1 = 0.0332, wR_2 = 0.0861	R_1 = 0.0313, wR_2 = 0.0663	R_1 = 0.0291, wR_2 = 0.0755
**Final R indexes [all data]**	R_1 = 0.0345, wR_2 = 0.0869	R_1 = 0.0459, wR_2 = 0.0755	R_1 = 0.0330, wR_2 = 0.0791
**Largest diff. peak/hole / e·Å³**	2.31/−1.81	1.86/−1.32	2.55/−1.81

Table 2. Crystallographic data.

Compound	$Hg(O_2CC_3H_7)$
Empirical formula	$C_8H_{14}HgO_4$
Formula weight	372.78
Temperature/K	295
Crystal system	triclinic
Space group	$P\bar{1}$
a/Å	4.6153(7)
b/Å	8.5983(14)
c/Å	13.661(2)
α/°	97.157(6)
β/°	99.537(5)
γ/°	102.882(5)
Volume/Å³	513.76(14)
Z	2
ρ_{calc}/g·cm³	2.423
μ/mm⁻¹	14.961
F(000)	348.0
Crystal size/mm³	0.871 x 0.531 x 0.381
Crystal shape	Needle
Radiation	MoKα (λ = 0.71073)
2Θ range for data collection/°	4.93 to 56.89
Index ranges	-6 ≤ h ≤ 6, -11 ≤ k ≤ 11, -18 ≤ l ≤ 18
Reflections collected	28054
Independent reflections	2577 [R_{int} = 0.0433, R_{sigma} = 0.0206]
Data/restrains/parameters	2577/0/123
Goodness-of-fit on F^2	1.120
Final R indexes [I>=2σ(I)]	R_1 = 0.0233, wR_2 = 0.0521
Final R indexes [all data]	R_1 = 0.0373, wR_2 = 0.0596
Largest diff. peak/hole / e·Å³	1.55/−0.75

Single crystals of $Hg(OOCC_3H_7)_2$ were obtained by crystallization from a hot solution in 1,2-dichloroethane. The compound crystallizes in triclinic space group $P\bar{1}$. The asymmetric unit contains two $Hg(OCC_3H_7)$ fragments. Due to a center of inversion located at both mercury atoms the overall formula is $Hg(O_2CC_3H_7)_2$. The mercury atoms are coordinated by 4 different carboxylic groups forming two short Hg-O bonds (2.054(4), 2.056(4) Å) in a linear fashion (O-Hg-O: 180°) and six weaker Hg-O contacts (2.680(4) – 3.086(4) Å) within the sum of the van-der-Waals radii of mercury[11] and oxygen,[12] respectively. Therefore, the coordination number for both metal centers is eight. The carboxylic groups are connecting up to three different mercury atoms yielding a polymeric network. Hg-Hg contacts shorter than twice the van-der-Waals radius of mercury are not observed. So far only a few examples of mercury complexes with a coordination number of eight are reported in the literature.[13–15]

$FeC_{10}(HgO_2CCF_3)_{10}$ · 4 THF · 2 OEt_2

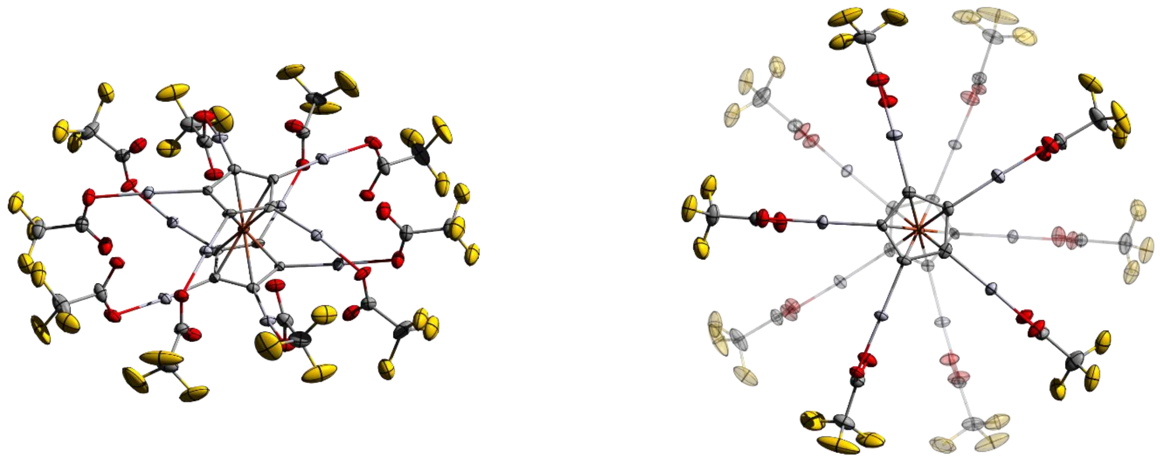

Figure S1. Molecular structure of $FeC_{10}(HgO_2CCF_3)_{10}$ · 4 THF · 2 OEt_2 in the solid state. Solvent molecules and distorted atoms are omitted for clarity. Ellipsoids (50% probability level). Left: front view. Right: top view. The second cyclopentadienyl ligand is drawn in a transparent fashion. Color code: light grey – mercury, orange – iron, yellow – fluorine, red – oxygen, grey – carbon.

$FeC_{10}(HgO_2CCCl_3)_{10}$ · 10 THF · OEt_2

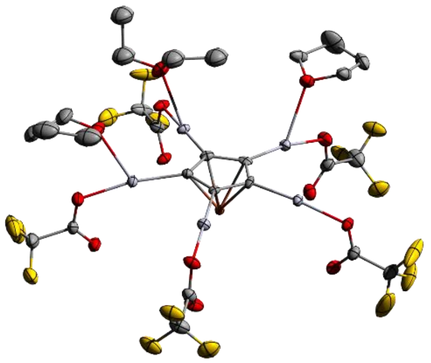

Figure S2. Asymmetric unit in the crystal structure of $FeC_{10}(HgO_2CCF_3)_{10}$ · 4 THF · 2 OEt_2. Hydrogen and distorted atoms are omitted for clarity. Ellipsoids (50% probability level). Color code: light grey – mercury, orange – iron, yellow – fluorine, red – oxygen, grey – carbon.

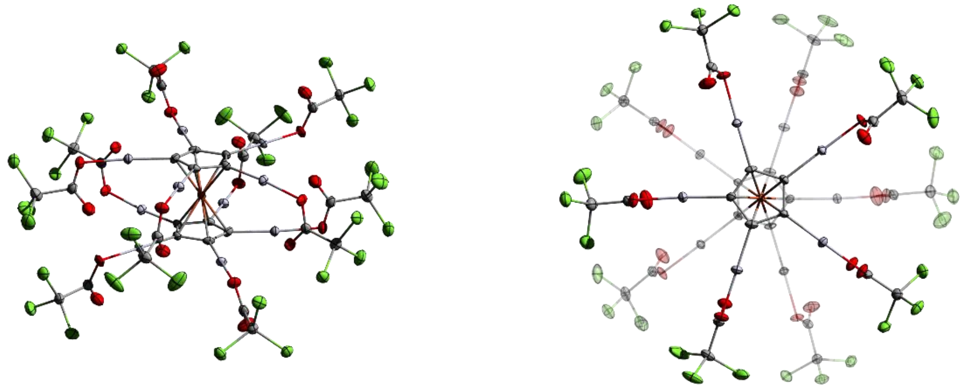

Figure S3. Molecular structure of $FeC_{10}(HgO_2CCCl_3)_{10}$ · 10 THF · OEt_2 in the solid state. Solvent molecules are omitted for clarity. Ellipsoids (50% probability level). Left: front view. Right: top view. The second cyclopentadienyl ligand is drawn in a transparent fashion. Color code: light grey – mercury, orange – iron, green – chlorine, red – oxygen, grey – carbon.

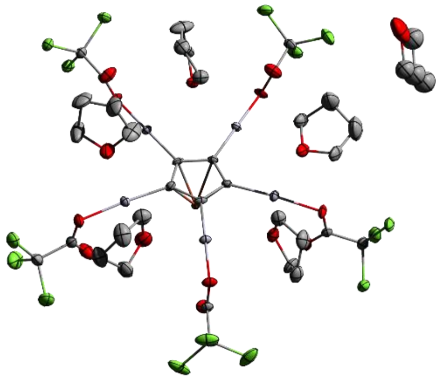

Figure 4. Asymmetric unit in the crystal structure of $FeC_{10}(HgO_2CCCl_3)_{10}$ · 10 THF · OEt_2. Hydrogen and distorted atoms are omitted for clarity. Ellipsoids (50% probability level). Color code: light grey – mercury, orange – iron, green – chlorine, red – oxygen, grey – carbon.

$FeC_{10}(HgCl)_{10}$ · 9 DMSO

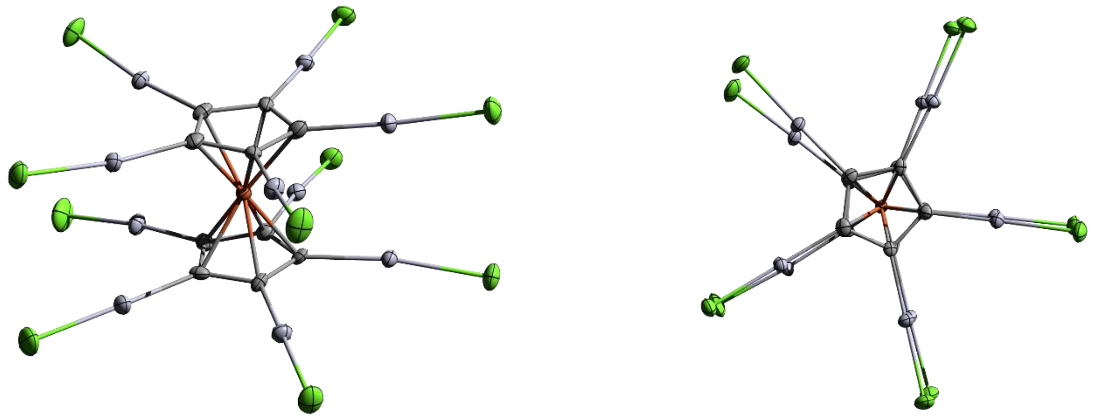

Figure S5. Molecular structure of $FeC_{10}(HgCl)_{10}$ · 9 DMSO in the solid state. Solvent molecules are omitted for clarity. Ellipsoids (50% probability level). Left: front view. Right: top view. Color code: light grey – mercury, orange – iron, green – chlorine, red – oxygen, grey – carbon.

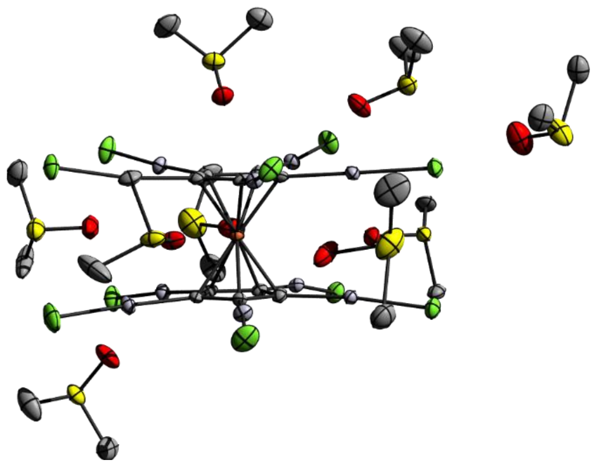

Figure 6. Asymmetric unit in the crystal structure of $FeC_{10}(HgCl)_{10}$ · 9 DMSO. Hydrogen and distorted atoms are omitted for clarity. Ellipsoids (50% probability level). Color code: light grey – mercury, orange – iron, green – chlorine, yellow – sulphur, red – oxygen, grey – carbon.

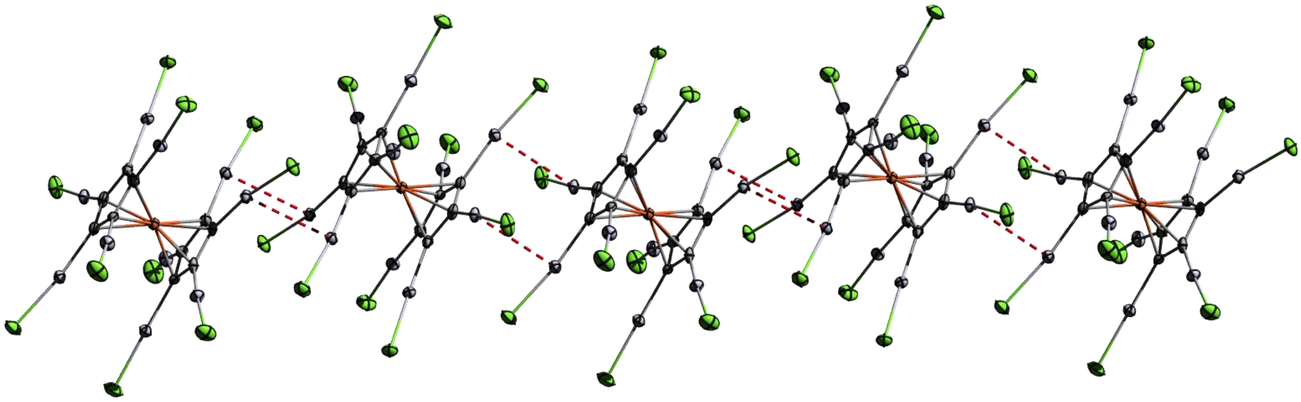

Figure S7. Crystal structure of $FeC_{10}(HgCl)_{10} \cdot 9$ DMSO. Front perspective. Ellipsoids (50% probability level). Solvent molecules are omitted for clarity. Color code: light grey – mercury, orange – iron, green – chlorine, grey – carbon.

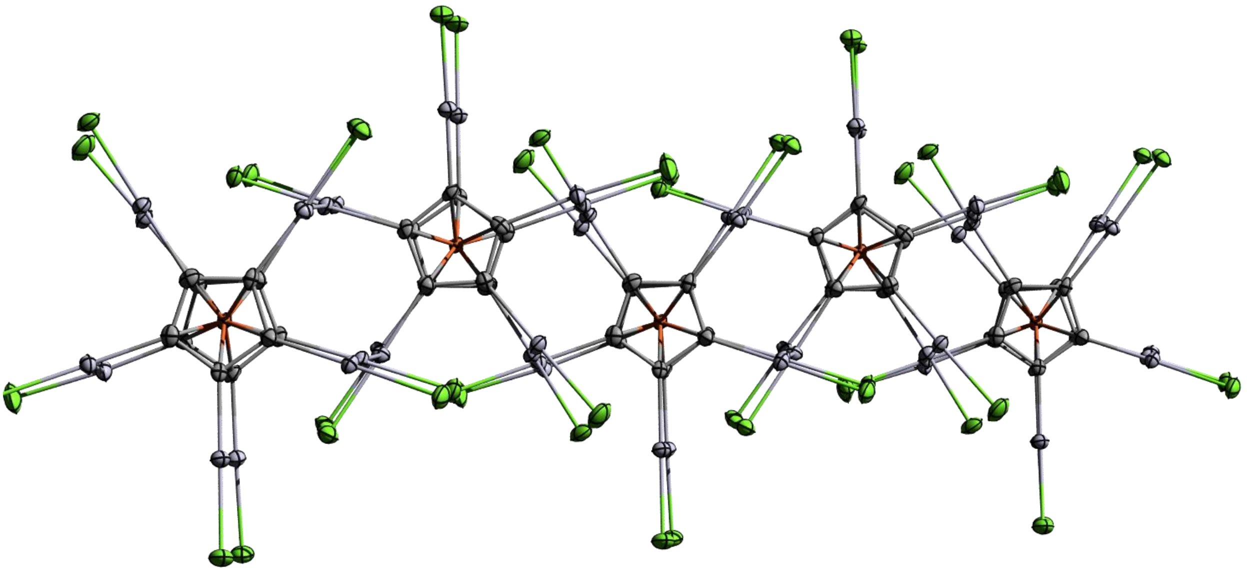

Figure S8. Crystal structure of $FeC_{10}(HgCl)_{10} \cdot 9$ DMSO. Top perspective. Ellipsoids (50% probability level). Solvent molecules are omitted for clarity. Color code: light grey – mercury, orange – iron, green – chlorine, grey – carbon.

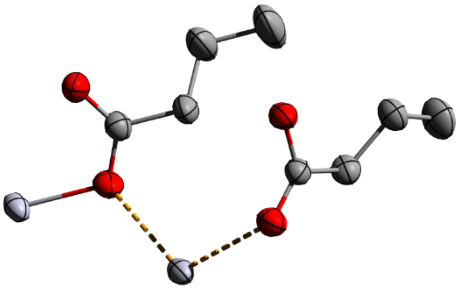

Figure S9. Asymmetric unit of the crystal structure of $Hg(O_2CC_3H_7)_2$. Hydrogen atoms are omitted for clarity. Ellipsoids (50% probability level). Color code: light grey – mercury, red – oxygen, grey – carbon.

References

[1] R. K. Harris, E. D. Becker, S. M. C. de Menezes, P. Granger, R. E. Hoffmann, K. W. Zilm, *Pure Appl. Chem.* **2008**, *80*, 59.
[2] N. T. H. J., S. M., *PLoS One* **2012**, *7*, e44913.
[3] M. R. Willcott, *J. Am. Chem. Soc.* **2009**, *131*, 13180.
[4] OriginLab, *No Title*, OriginLab Corp., Northhampton, Massachusetts, USA, **2016**.
[5] O. V. Dolomanov, L. J. Bourhis, R. J. Gildea, J. A. K. Howard, H. Puschmann, *J. Appl. Cryst.* **2009**, *42*, 339–341.
[6] G. M. Sheldrick, *Acta Cryst.* **2015**, *A71*, 3–8.
[7] G. M. Sheldrick, *SHELXL Version 2014/7, Program for Chrystal Structure Solution and Refinement*, Göttingen, Germany, **2014**.
[8] G. M. Sheldrick, *Acta Cryst.* **2008**, *A64*, 112–122.
[9] K. Brandenburg, “Diamond: Crystal and Molecular Structure Visualization,” can be found under http://www.crystalimpact.com/diamond, **2017**.
[10] Persistance of Version Pty. Ltd., **2004**, Retrieved from http://www.povray.org/download/.
[11] P. Pyykkö, M. Straka, *Phys. Chem. Chem. Phys.* **2000**, *2*, 2489–2493.
[12] A. Bondi, *J. Phys. Chem.* **1964**, *68*, 441–451.
[13] N. J. Williams, R. D. Hancock, J. H. Riebenspies, M. A. Fernandes, A. S. de Sousa, *Inorg. Chem.* **2009**, *48*, 11724–11733.
[14] C. Kimblin, V. J. Murphy, T. Hascall, B. M. Bridgewater, J. B. Bonanno, G. Parkin, *Inorg. Chem.* **2000**, *39*, 967–974.
[15] J. Halfpenny, R. W. H. Small, *Acta Crystallorgr., Sect. C Cryst. Struct. Commun.* **1997**, *53*, 438–443.

Appendix

Spectra

Bis(butyryloxy)mercury(II)

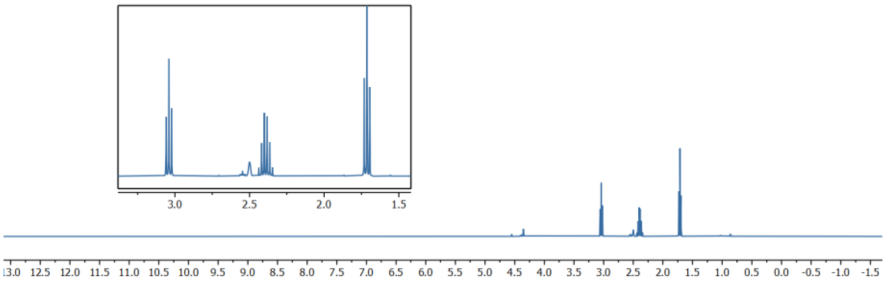

Figure S10. ^{1}H NMR spectrum of bis(butyryloxy)mercury(II) (400 MHz, dmso-d_6, r.t.).

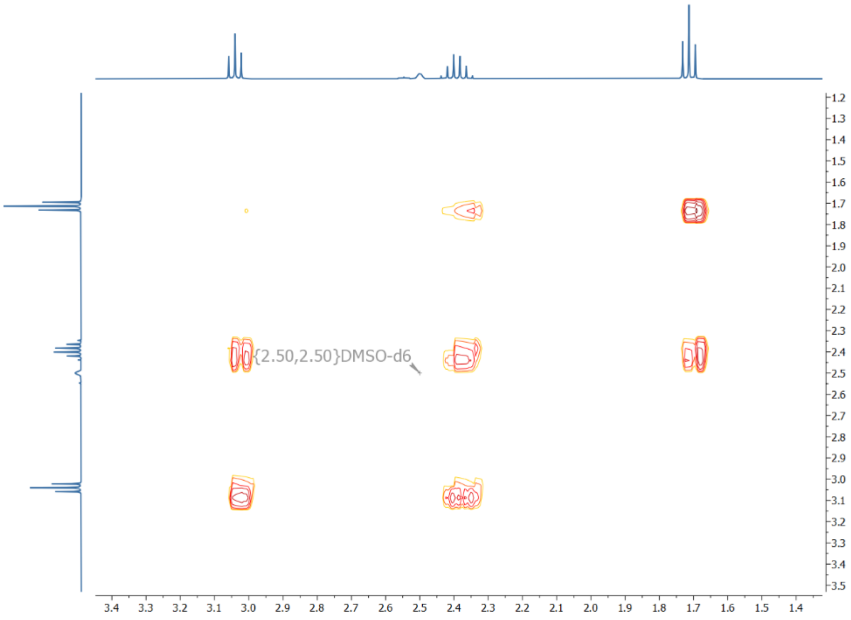

Figure S11. $^{1}H,^{1}H$ COSY NMR spectrum (400 MHz, dmso-d_6, r.t.).

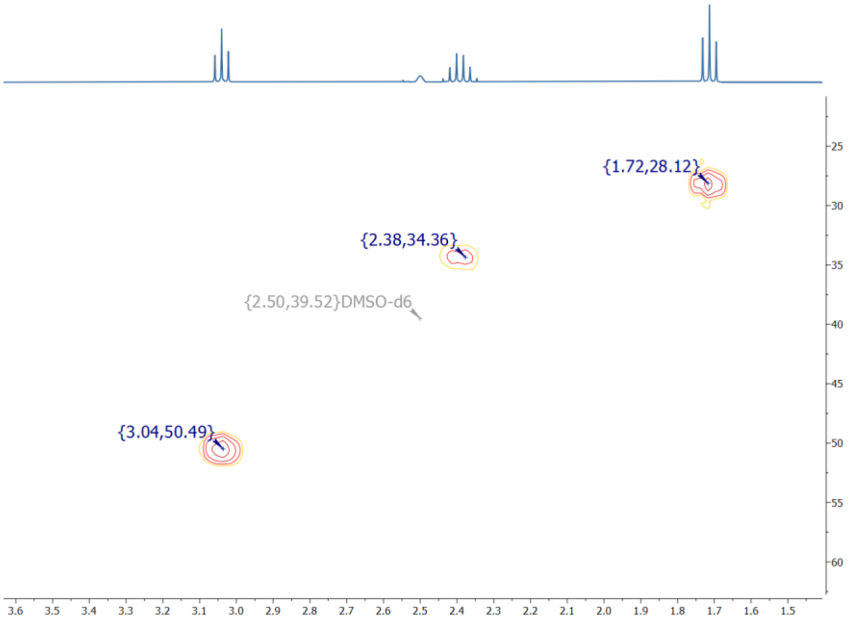

Figure S12. $^{1}H,^{13}C$ HMQC NMR spectrum (400 MHz, dmso-d_6, r.t.).

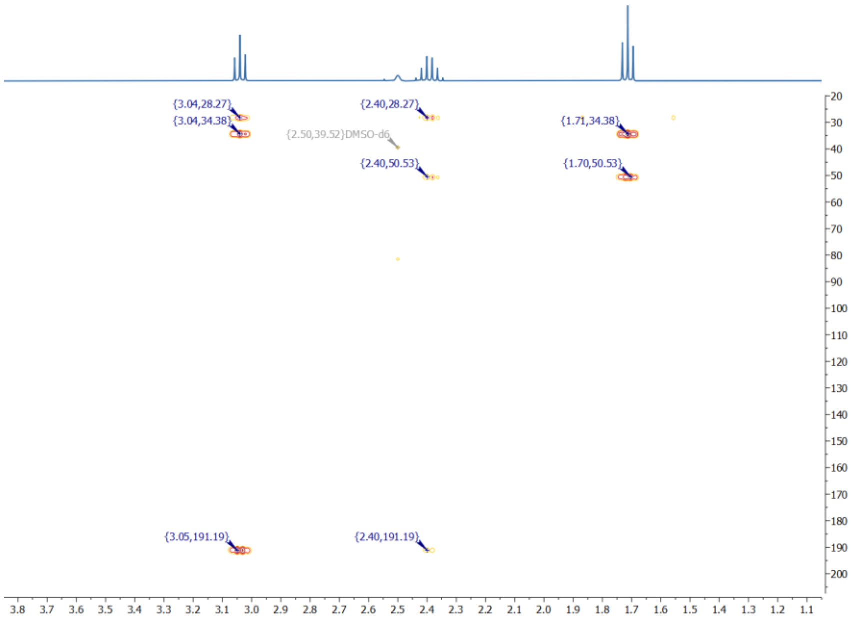

Figure S13. $^{1}H,^{13}C$ HMBC NMR spectrum (400 MHz, dmso-d_6, r.t.).

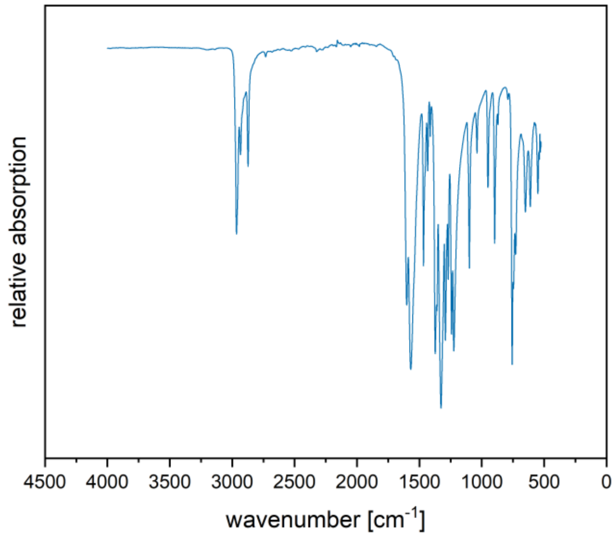

Figure S14. Infrared spectrum (ATR) of compound $Hg(O_2CC_3H_7)_2$.

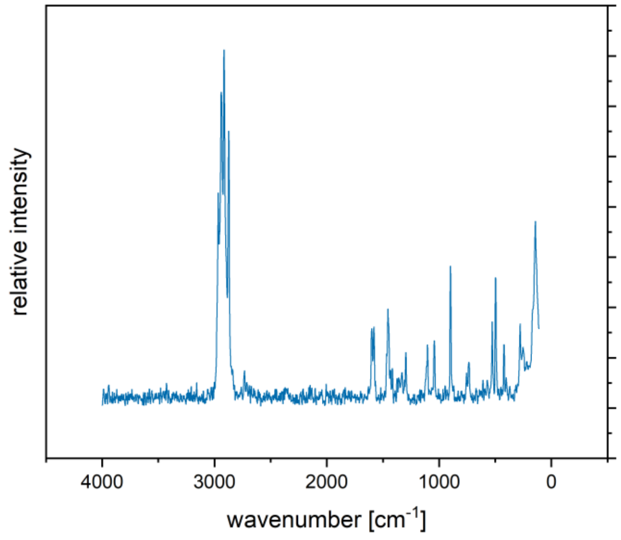

Figure S15. Raman spectrum (1064 nm) of $Hg(O_2CC_3H_7)_2$.

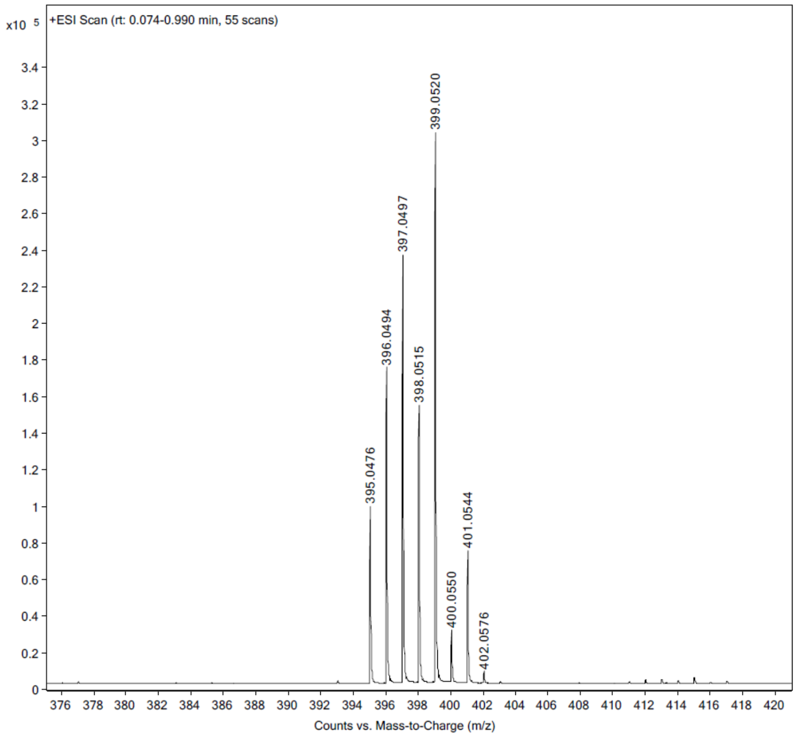

Figure S16. Mass spectrum of $Hg(O_2CC_3H_7)_2$, RT, ESI+, DCM/MeOH.

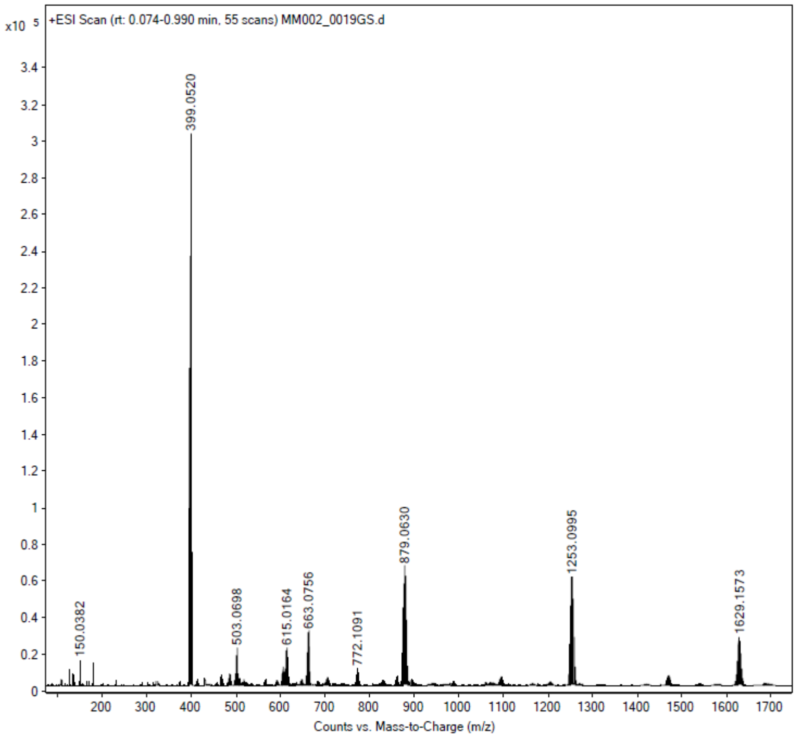

Figure S17. Mass spectrum of $Hg(O_2CC_3H_7)_2$, RT, ESI+, DCM/MeOH.

Decakis(butyryloxymercury(II))ferrocene ***2***

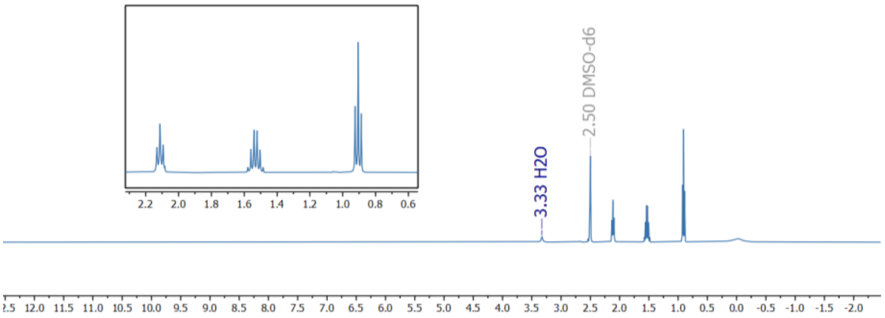

Figure S19. ^{1}H NMR spectrum of $FeC_{10}(HgO_2CC_3H_7)_{10}$ (700 MHz, dmso-d_6, r.t.).

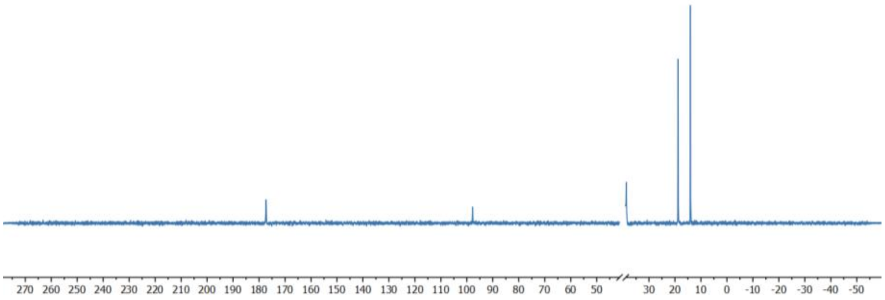

Figure S20. ^{13}C NMR spectrum of $FeC_{10}(HgO_2CC_3H_7)_{10}$ (176 MHz, dmso-d_6, r.t.). The signal of the deuterated solvent is omitted for clarity.

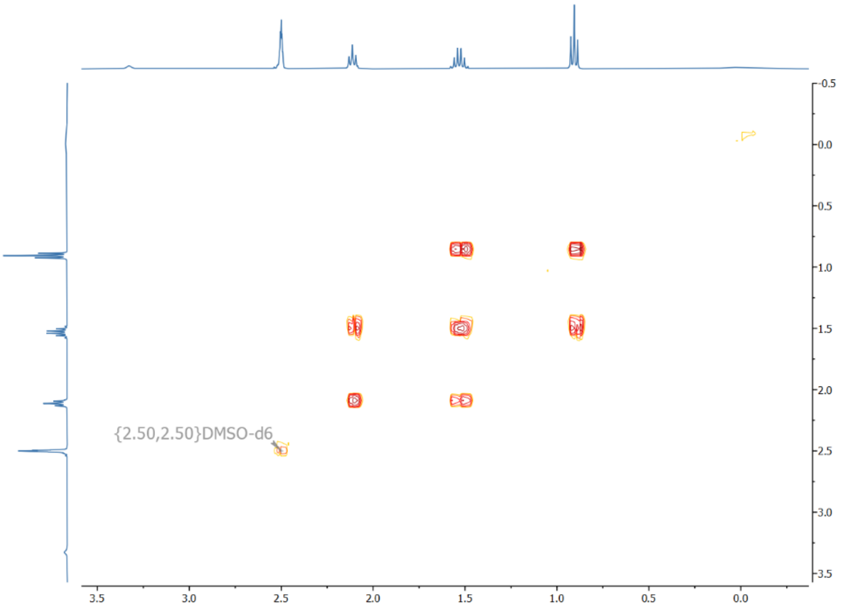

Figure S18. ^{1}H,^{1}H COSY NMR spectrum of $FeC_{10}(HgO_2CC_3H_7)_{10}$ (700 MHz, dmso-d_6, r.t.).

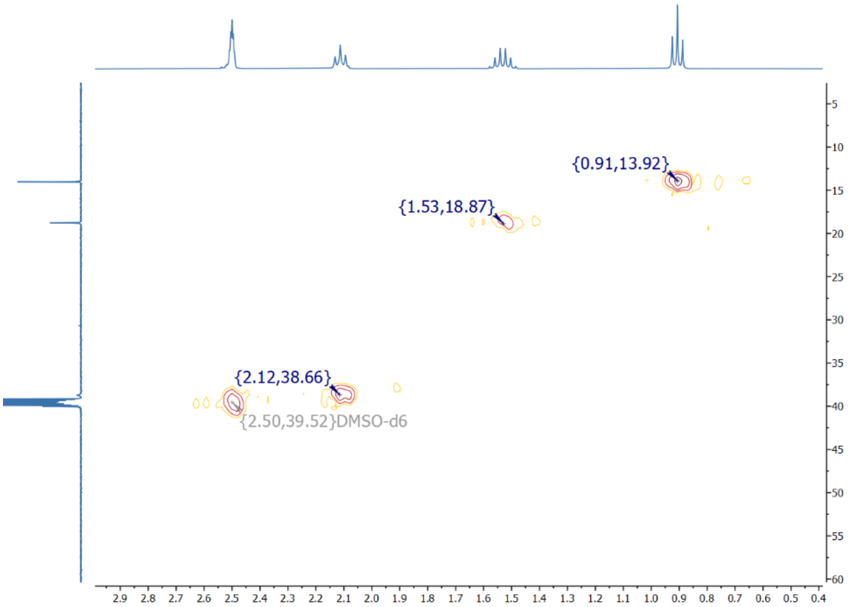

Figure S21. $^{1}H,^{13}C$ HMQC NMR spectrum of $FeC_{10}(HgO_2CC_3H_7)_{10}$ (700 MHz, dmso-d_6, r.t.).

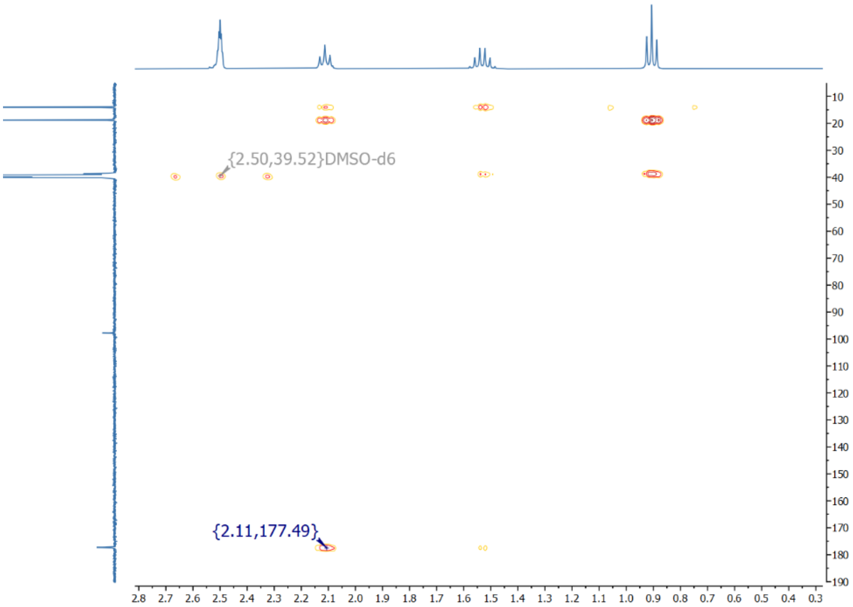

Figure S22. $^{1}H,^{13}C$ HMBC NMR spectrum of $FeC_{10}(HgO_2CC_3H_7)_{10}$ (700 MHz, dmso-d_6, r.t.).

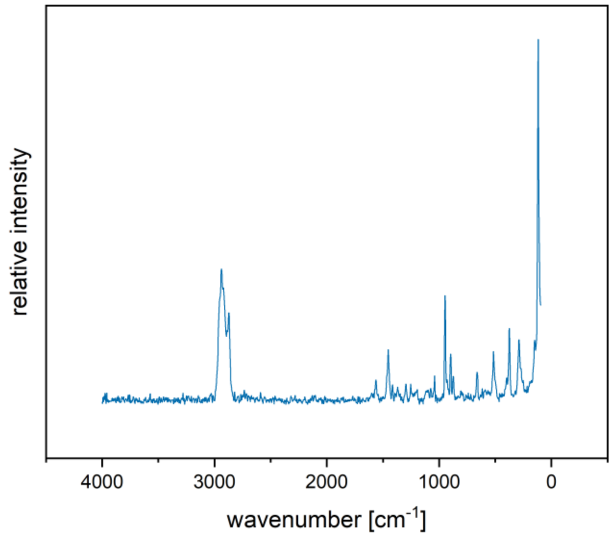

Figure S23. Raman spectrum of $FeC_{10}(HgO_2CC_3H_7)_{10}$ (1064 nm, r.t.).

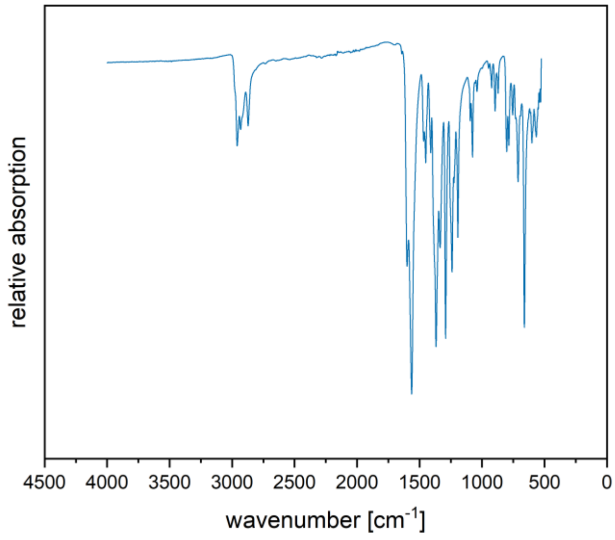

Figure S24. Infrared spectrum (ATR) of compound $FeC_{10}(HgO_2CC_3H_7)_{10}$.

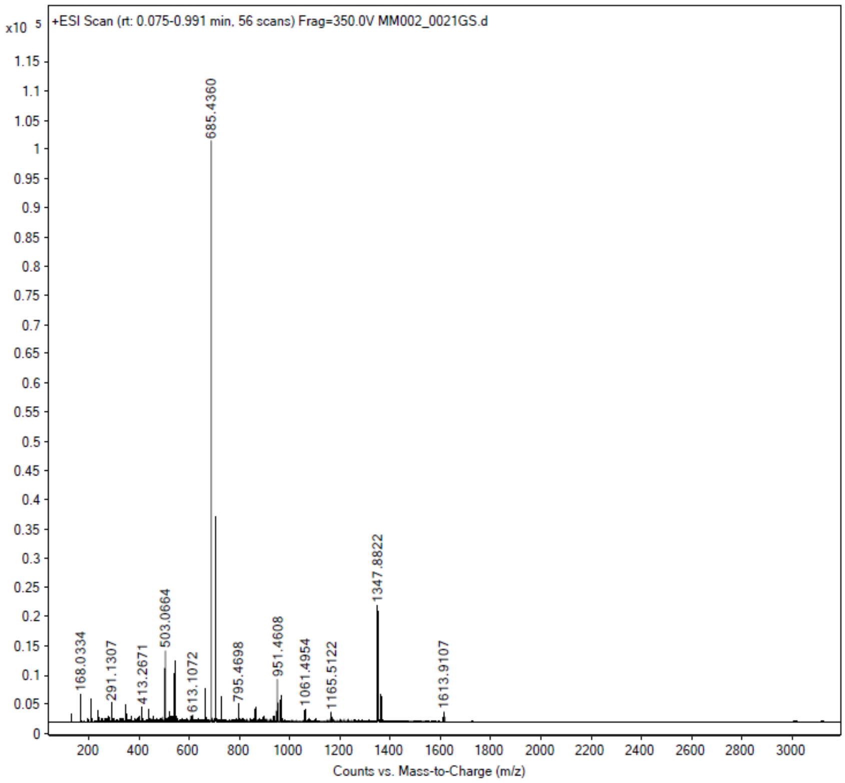

Figure S25. Mass spectrum of $FeC_{10}(HgO_2CC_3H_7)_{10}$, RT, ESI+, DCM/MeOH.

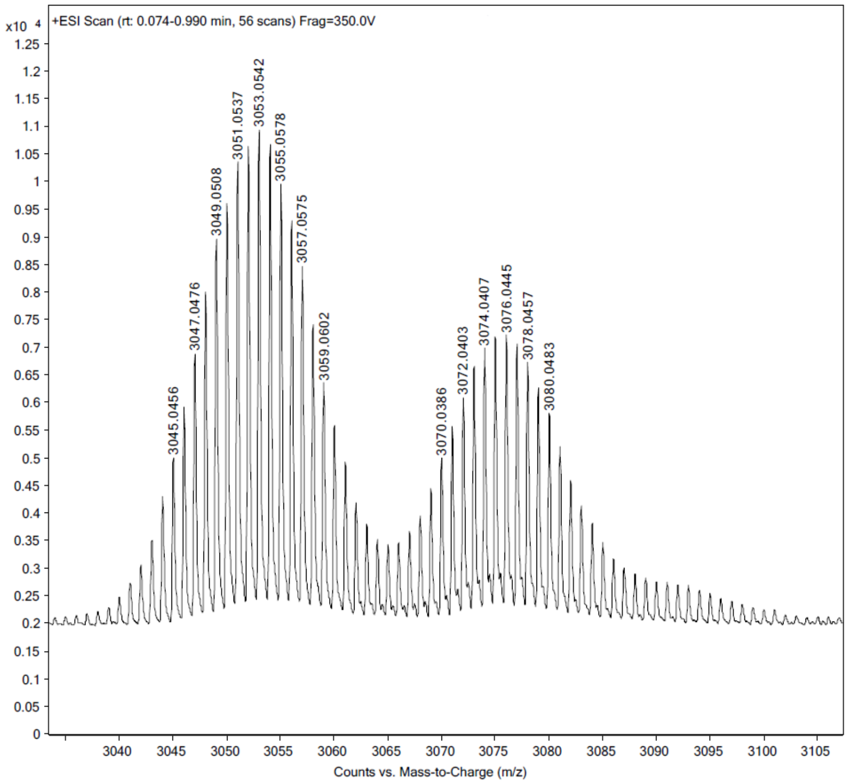

Figure S26. Mass spectrum of $FeC_{10}(HgO_2CC_3H_7)_{10}$, RT, ESI+, DCM/MeOH.

Decakis(trifluoroacetoxymercury(II))ferrocene

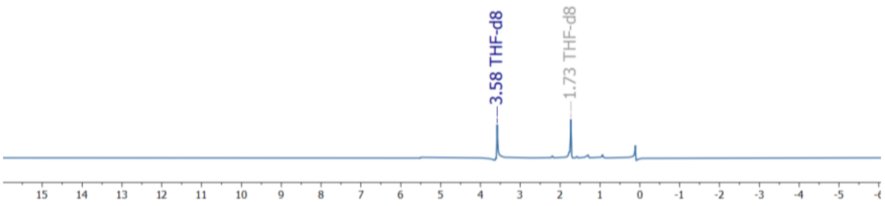

Figure S27. ^{1}H NMR spectrum of $FeC_{10}(HgO_2CCF_3)_{10}$ (700 MHz, THF-d_8, r.t.).

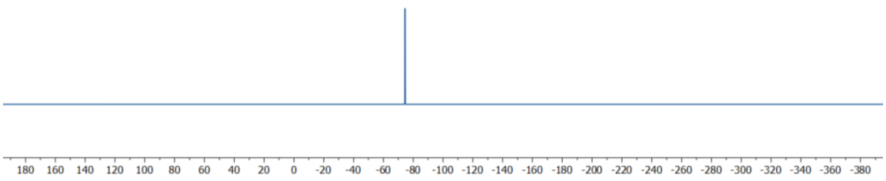

Figure S28. ^{19}F NMR spectrum of $FeC_{10}(HgO_2CCF_3)_{10}$ (564 MHz, THF-d_8, r.t.).

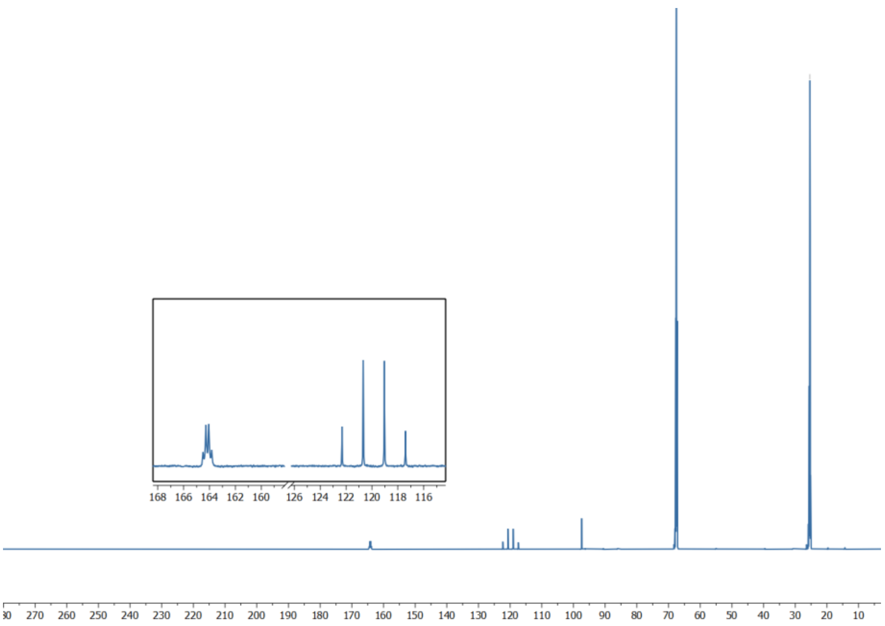

Figure S29. $^{13}C\{^{1}H\}$ NMR spectrum of $FeC_{10}(HgO_2CCF_3)_{10}$ (176 MHz, THF-d_8, r.t.).

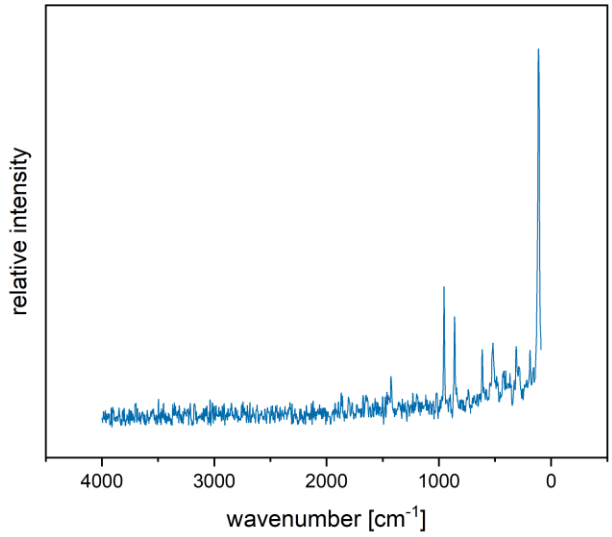

Figure S30. Raman spectrum of of $FeC_{10}(HgO_2CCF_3)_{10}$ (1064 nm, r.t.).

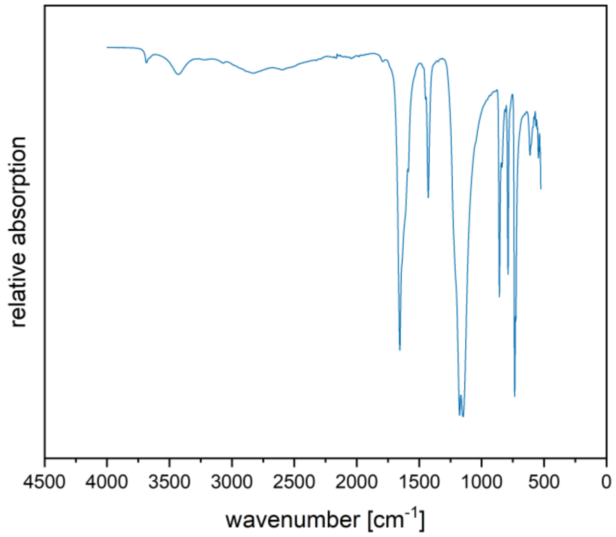

Figure S31. Infrared spectrum (ATR) of compound $FeC_{10}(HgO_2CCF_3)_{10}$.

Decakis(trichloroacetoxymercury(II))ferrocene

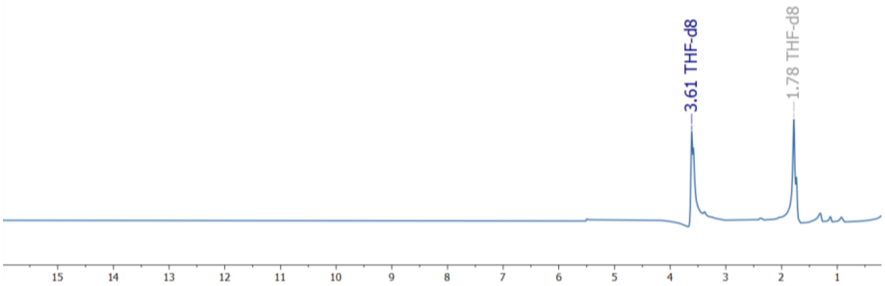

Figure S32. ^{1}H NMR spectrum of $FeC_{10}(HgO_2CCCl_3)_{10}$ (700 MHz, THF-d_8, r.t.).

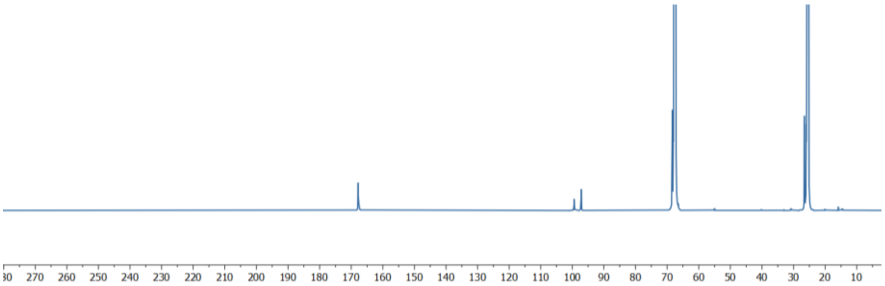

Figure S33. ^{13}C NMR spectrum of $FeC_{10}(HgO_2CCCl_3)_{10}$ (176 MHz, THF-d_8, r.t.).

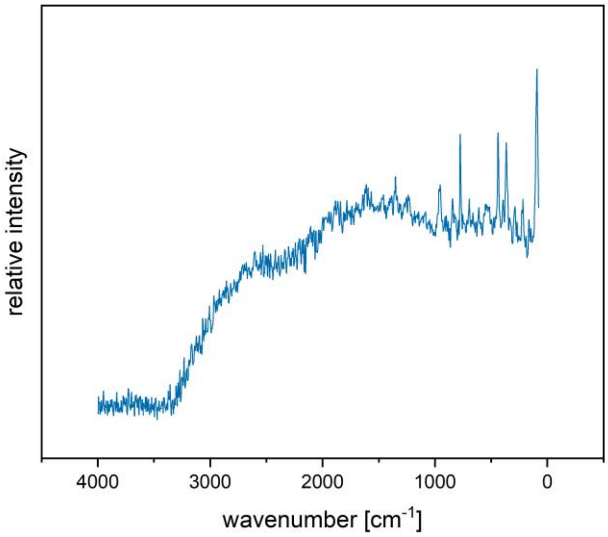

Figure 34. Raman spectrum (1064 nm) of $FeC_{10}(HgO_2CCCl_3)_{10}$

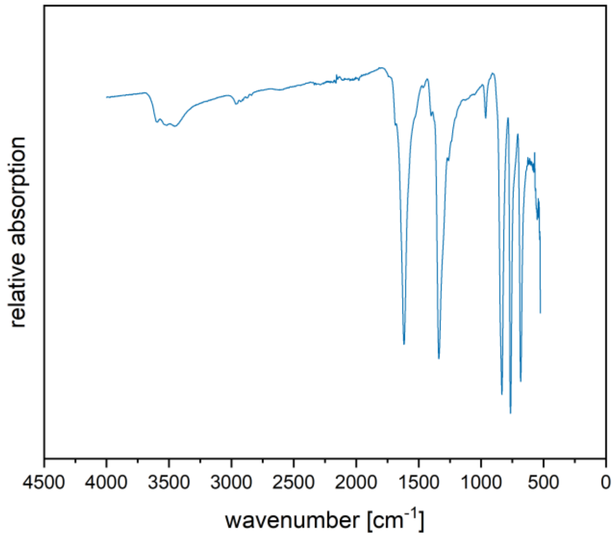

Figure S35. Infrared spectrum (ATR) of compound $FeC_{10}(HgO_2CCCl_3)_{10}$.

Decakis(fluoridomercury)ferrocene

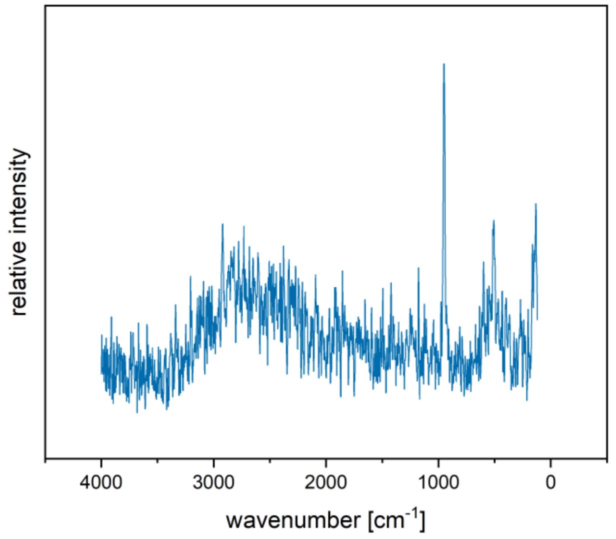

Figure S36. Raman spectrum (1064 nm) of $FeC_{10}(HgF)_{10}$.

Decakis(chloridomercury)ferrocene

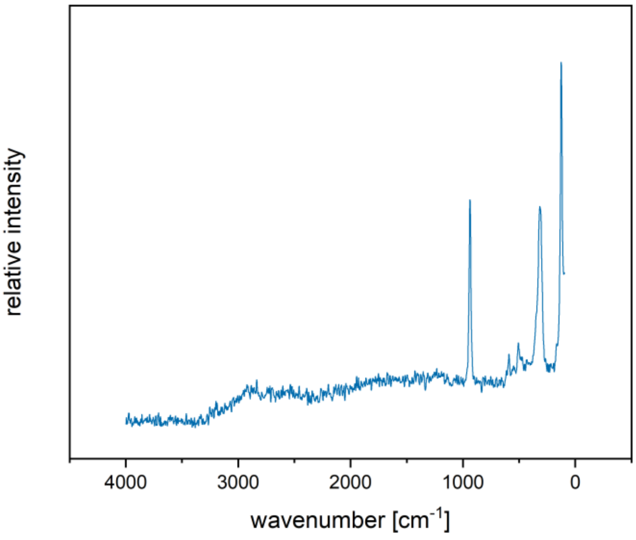

Figure S37. Raman spectrum (1064 nm) of $FeC_{10}(HgCl)_{10}$.

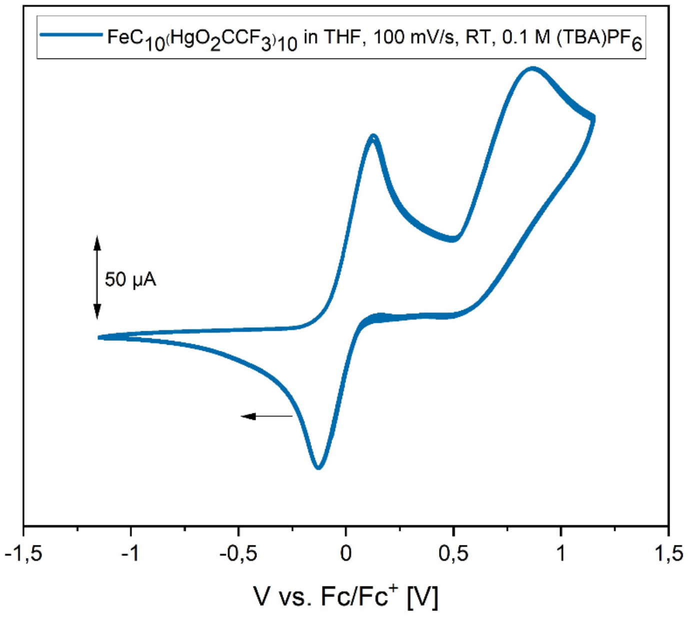

Figure S38. Cyclic voltammogram of $FeC_{10}(HgO_2CCF_3)_{10}$ referenced vs. Cp_2Fe.
